# Integrin αvβ3 Is a Master Regulator of Resistance to TKI-Induced Ferroptosis in HER2-Positive Breast Cancer

**DOI:** 10.3390/cancers15041216

**Published:** 2023-02-14

**Authors:** Aadya Nagpal, Kristen Needham, Darius J. R. Lane, Scott Ayton, Richard P. Redvers, Melissa John, Heloisa S. Selistre-de-Araujo, Delphine Denoyer, Normand Pouliot

**Affiliations:** 1 Matrix Microenvironment & Metastasis Laboratory, Olivia Newton-John Cancer Research Institute, Heidelberg, VIC 3084, Australia; 2 School of Cancer Medicine, La Trobe University, Bundoora, VIC 3086, Australia; 3 Tumour Angiogenesis and Microenvironment Laboratory, Peter MacCallum Cancer Centre, Melbourne, VIC 3000, Australia; 4 Sir Peter MacCallum Department of Oncology, The University of Melbourne, Melbourne, VIC 3000, Australia; 5 Oncogenic Transcription Laboratory, Olivia Newton-John Cancer Research Institute, Heidelberg, VIC 3084, Australia; 6 The Florey Institute of Neuroscience and Mental Health, Parkville, VIC 3052, Australia; 7 Metastasis Research Laboratory, Olivia Newton-John Cancer Research Institute, Heidelberg, VIC 3084, Australia; 8 Department of Physiological Sciences, Center of Biological and Health Science, Federal University of São Carlos, São Carlos 13565-905, SP, Brazil; 9 Department of Clinical Pathology, The University of Melbourne, Melbourne, VIC 3000, Australia

**Keywords:** HER2-positive breast cancer, drug resistance, ferroptosis, tyrosine kinase inhibitors, cell adhesion receptors, αvβ3 integrin

## Abstract

**Simple Summary:**

Intrinsic or acquired resistance to clinically approved targeted therapies for breast cancer patients is a major cause of treatment failure. Our findings provide conclusive functional evidence that the cell adhesion receptor αvβ3 integrin is a critical mediator of resistance to Human Epidermal Growth Factor Receptor -2 (HER2)-targeting small molecule tyrosine kinase inhibitors (TKIs). We show that αvβ3 integrin contributes to the persistent activation of the AKT signalling pathway as well as to the re-wiring of the iron and antioxidant metabolism of the cells, thereby providing increased protection from an iron-dependent form of cell death called ferroptosis. Importantly, we demonstrate that genetic manipulation or therapeutic targeting of this receptor provides a novel strategy to reverse the resistance to TKI-induced ferroptosis in mouse and human HER2-positive breast cancer cells. We propose that the increased dependency on αvβ3 integrin signalling in TKI-resistant cells represents an Achilles’ heel that can be targeted pharmacologically to improve patient outcomes.

**Abstract:**

Human epidermal growth factor receptor-2 (HER2)-targeting therapies provide clinical benefits for patients with HER2-positive breast cancer. However, the resistance to monotherapies invariably develops and leads to disease relapse and treatment failure. Previous studies have demonstrated a link between the potency of HER2-targeting tyrosine kinase inhibitors (TKIs) and their ability to induce an iron-dependent form of cell death called ferroptosis. The aim of this study was to understand the mechanisms of resistance to TKI-induced ferroptosis and identify novel approaches to overcome treatment resistance. We used mouse and human HER2-positive models of acquired TKI resistance to demonstrate an intimate link between the resistance to TKIs and to ferroptosis and present the first evidence that the cell adhesion receptor αvβ3 integrin is a critical mediator of resistance to TKI-induced ferroptosis. Our findings indicate that αvβ3 integrin-mediated resistance is associated with the re-wiring of the iron/antioxidant metabolism and persistent activation of AKT signalling. Moreover, using gene manipulation approaches and pharmacological inhibitors, we show that this “αvβ3 integrin addiction” can be targeted to reverse TKI resistance. Collectively, these findings provide critical insights into new therapeutic strategies to improve the treatment of advanced HER2-positive breast cancer patients.

## 1. Introduction 

Approximately 15–20% of breast cancers present with an upregulation of the human epidermal growth factor receptor-2 (HER2), forming an aggressive tumour subtype with a high propensity to metastasise to distant organs, including to the brain [1,2]. Over the last two decades, there have been immense developments in the landscape of HER2-targeting therapies, with eight anti-HER2 agents currently approved by the Food and Drug Administration (FDA). These include monoclonal antibodies targeting HER2 extracellular domains (trastuzumab, pertuzumab, and margetuximab); antibody–drug conjugates (ADCs) that combine HER2-targeting antibodies with cytotoxic agents (ado-trastuzumab-emtansine (TDM1) and trastuzumab deruxtecan (DS-8201a)); and small molecule tyrosine kinase inhibitors (TKIs) that target the intracellular HER kinase domain (lapatinib, neratinib, and tucatinib) [3,4]. The current standard of care for patients diagnosed with metastatic HER2-positive breast cancer consists of dual targeting with anti-HER2 antibodies (trastuzumab, pertuzumab) and a-taxane chemotherapy [5]. TDM-1 is offered as a second-line therapy for patients who relapse <12 months after treatment [6]. Despite the clinical benefit of an antibody-based regimen, less than 35% of patients initially respond to trastuzumab and for those who do respond, 70% develop secondary or acquired resistance and progress to metastatic disease within a year [7]. Moreover, due to heterogeneity in the blood–brain barrier permeability, antibody-based therapies show limited benefits against CNS metastases, which become a major cause of mortality in these patients [8,9,10]. Owing to their small size, their demonstrated activity in trastuzumab-resistant cells/tumours and their ability to target multiple EGFR family receptors, TKIs represent attractive therapeutic agents to treat residual disease after dual antibody-based HER2-targeting therapy [11]. However, the partial efficacy of TKI monotherapies reported in clinical trials indicate that resistance to treatment can still develop, leading to disease relapse and an increased incidence of incurable brain metastases [12,13]. Hence, developing new strategies to improve the efficacy and/or overcome resistance to TKIs in patients with advanced HER2-positive breast cancer is a clinical priority. 

Ferroptosis is an iron-dependent cell-death process associated with the accumulation of reactive oxygen species (ROS) and lipid peroxidation that compromises the integrity of cellular membranes [14]. In general terms, ferroptosis is induced by conditions that alter the balance between iron and antioxidant/redox metabolism and lead to excessive oxidative stress. Canonically, ferroptosis involves the inactivation/suppression of glutathione peroxidase 4 (GPX4) whose role is to protect biomembranes from phospholipid peroxidation [15]. GPX4 activity depends on the availability of the intracellular antioxidant, glutathione (GSH), whose synthesis requires the import of extracellular cystine by System Xc- (SLC3A2/SLC7A11 dimer) [16]. Accordingly, ferroptosis can be induced in cells by inhibitors of GPX4 (e.g., RSL3), GSH synthesis (e.g., buthionine sulphoximine, BSO) or System Xc- (e.g., Erastin) [17,18,19]. Ferroptosis can also be initiated by an increased labile iron/Fe2+ pool and regulated by the iron importer transferrin receptor 1 (TFR-1), iron storage protein, ferritin, and iron exporter, ferroportin-1 [20]. The activation of autophagy pathways that induce the degradation of ferritin (ferritinophagy) [21] or inhibition of ferroportin-1 also promotes ferroptosis [22]. 

We and others have shown that TKIs differ in their ability to trigger ferroptosis [23,24,25]. The sensitivity of triple-negative breast cancer cells to EGFR-targeting TKI gefitinib has been shown to correlate with the induction of ferroptotic cell death [26]. EGFR and HER2-targeting TKI lapatinib, while insufficient alone, sensitises cells to ferroptosis inducers [23,24]. Importantly, our previous findings provided the first evidence that, unlike lapatinib or tucatinib, the superior activity of pan-HER-targeting neratinib against mouse and human HER2-positive tumour lines in vitro and brain metastases in vivo is associated with neratinib’s unique ability to directly induce cell death by ferroptosis [23]. However, the residual disease detected in the lung/liver of some neratinib-treated mice and a lack of response against the late-stage metastatic disease indicate that the acquired resistance can develop [23]. Thus, to implement effective pro-ferroptotic TKI-based therapies in the clinic, it is critical to clarify how resistance develops. 

Extracellular matrix (ECM) proteins and cell adhesion integrin receptors have been implicated in mediating protection from anti-cancer therapies, including from TKIs [27]. In particular, α6β4, αvβ3, and β1-type integrins have been shown to contribute to breast cancer metastasis to the CNS [28,29,30] and to other organs [31,32,33,34], to regulate cancer stem-cell phenotypes [35,36] and to mediate the resistance to systemic chemotherapy and EGFR/HER2-targeting therapies [27,37,38]. However, the field is divided on the dominant integrin receptor regulating therapy resistance in HER2-positive breast cancer, and the precise molecular mechanisms involved remain poorly characterised. Furthermore, whether integrin targeting is a viable strategy to prevent or reverse resistance to TKI-induced ferroptosis, particularly in the context of HER2-positive breast cancer, has not been explored. 

Here, we developed and characterised mouse and human HER2-positive models that are resistant to neratinib treatment. Findings in these models show that the resistance to neratinib-induced ferroptosis is associated with cross-resistance to multiple TKIs and to ferroptosis inducers, acquisition of a mesenchymal-like morphology, and upregulation of cell surface αvβ3 integrin receptor. Importantly, we show that genetic deletion of β3 integrin or pharmacological inhibition of this receptor reverses resistance to neratinib-induced ferroptosis and increases sensitivity to other TKIs and to ferroptosis inducers. These effects can be reversed by forced overexpression of β3 integrin in cells lacking or naturally expressing low levels of β3 integrin. Mechanistically, β3 integrin was found to promote persistent AKT signalling in resistant cells and to reprogram iron and antioxidant metabolism. Hence, our findings establish αvβ3 integrin as a key regulator of TKI resistance in HER2-positive breast cancer and a relevant therapeutic target to reverse resistance to TKI-induced ferroptosis.

## 2. Materials and Methods 

### 2.1. Cell Culture and Reagents 

Murine HER2-positive brain metastatic TBCP-1 cells and human non-metastatic HER2-positive BT474 and SKBR3 cells were cultured as previously described [23]. Human brain metastatic HER2-positive cells SUM190Br, JIMT1Br3, and MCF7-HER2Br3 were kindly provided by Dr. Patricia Steeg (Centre for Cancer Research, National Cancer Institute, Bethesda, MA, USA) and were cultured as previously described [39,40]. Human embryonic kidney 293 expressing a temperature-sensitive allele of the SV40 T antigen (HEK293T) was used for lentiviral production and cultured in Dulbecco’s modified Eagle’s medium (DMEM) supplemented with 10% (v/v) foetal bovine serum (FBS) and 1% penicillin/streptomycin (P/S).

Neratinib-resistant variants of mouse and human HER2-positive cell lines (TBCP-1NR, BT474NR and SKBR3NR) were generated by long-term culture of the respective cell lines in increasing concentrations of neratinib (up to 2 µM). Resistant variants were maintained in the same media as age-matched sensitive control cells. All cell lines were maintained in a humidified incubator at 37 °C under 5% CO_2_. For routine culture and experimentation, cells were passaged when subconfluent and kept in culture for a maximum of 4 weeks. 

Lapatinib ditosylate, tucatinib hydrochloride, and RAS synthetic lethal 3 (RSL3) were obtained from SelleckChem (Scorseby, VIC, Australia). Erastin and liproxstatin-1 were purchased from Sigma-Aldrich (Castle Hill, NSW, Australia). Neratinib maleate was provided by Puma Biotechnology (Los Angeles, CA, USA). AKT inhibitor VIII was purchased from Calbiochem (San Diego, CA, USA). Cilengitide was purchased from Mimotopes (Mulgrave, VIC, Australia). All compounds were prepared at 5–10 mM stocks in DMSO and diluted to the required concentration in the appropriate assay buffer immediately before in vitro and in vivo assays. pET28a-Disba plasmid, kindly provided by Dr. Heloisa Selistre-De-Araujo, was used for the production of recombinant Disba-01 as previously described in [41]. 

### 2.2. Stable Knockout (KO) of Integrin β3 or Ferroportin-1

Two predesigned guide RNAs (gRNAs) targeting integrin β3 or ferroportin-1 were purchased from Integrated DNA Technologies (Coralville, IA, USA). The gRNAs are conjugated with ATTO-550 fluorophore to aid with the selection of transfected cells. To form the targeting ribonucleoprotein (RNP) complex, each gRNA (1.5 µL) was combined with 1.5 µL of prediluted Cas9 enzyme at 1 µM, 0.6 µL of Cas9 Plus Reagent from CRISPRMAX and 21.4 µL of Opti-MEM medium. This complex was then combined with 1.2 µL of CRISPRMAX reagent and 23.8 µL of Opti-MEM medium to form the transfection complex, which was incubated at room temperature (RT) for 20 min. The target cells were detached using PBS containing 0.01% EDTA and re-suspended at 4 × 10^5^ cells/mL in FBS-containing media without antibiotics. The cell suspension (100 µL) was added to a well of a 96-well plate containing the transfection complex and incubated for 48 h at 37 °C. Successfully transfected cells were selected based on ATTO-550 positivity and single cells were sorted by FACS in 96-well plates. Single-cell clones were expanded and screened for lack of integrin β3 or ferroportin-1 expression by Western blotting.

### 2.3. Overexpression (OE) of Integrin β3 or Ferroportin-1

To generate control, integrin β3, or ferroportin-1 overexpressing cell lines, the following plasmids were cloned by Vector Builder (Chicago, IL, USA) according to our design: mouse empty vector control (pLV[Exp]-Puro-EF1A>{Stuffer_300bp}); mouse β3 integrin OE vector (pLV[Exp]-Puro-EF1A>mItgb3[NM_016780.2]); human empty vector control (pLV[Exp]-EF1A>ORF_Stuffer-CMV>tdTomato(ns):T2A:Puro); human β3 integrin OE vector (pLV[Exp]-EF1A>hITGB3[NM_000212.2]-CMV>tdTomato(ns):T2A:Puro); and human ferroportin-1 OE vector (pLV[Exp]-EF1A>hSLC40A1[NM_014585.6]-CMV>tdTomato(ns):T2A:Puro). These plasmids were received as E. Coli glycerol stocks and plated on ampicillin-containing plates to generate single-cell colonies for expansion and plasmid isolation. The plasmid DNA was extracted from bacterial pellets using Wizard^®^Plus SV Minipreps DNA Purification System kit (Promega, #A1330) as per the manufacturer’s instruction. For transfection, total plasmid DNA (2.5 μg) containing empty or integrin β3/ferroportin-1 expression vector and packaging vectors (pCMVΔR8.2, second-generation lentiviral plasmid and pCMV-VSV-G, envelop plasmid obtained form Adgene) in equimolar concentrations were added to 125 µL of Opti-MEM, followed by the addition of 5 µL of P3000 Lipofectamine Reagent. The plasmid mix was further combined with Lipofectamine 3000 Reagent pre-diluted in Opti-MEM in a 1:1 ratio and incubated at RT for 15 min to form a lipid-DNA complex that was added to the HEK293T cells with an additional 750 µL Opti-MEM. The transfected HEK293T cells were incubated for 24 h at 37 °C, 5% CO_2_. The transfection medium was then replaced with 1 mL of fresh DMEM supplemented with 10% FBS (without antibiotics) for another 24 h. The lentiviral supernatant was collected and filtered through a 0.45 µm filter. After the addition of polybrene (8 µg/mL), the lentiviral supernatant was added to semi-confluent target cancer cells for 24 h. Fresh complete DMEM (1 mL) was added to the transfected HEK293T cells and harvested after another 24 h for a second round of transduction as described above. Stably transduced cells were selected either by incubation with puromycin (5 µg/mL)-containing complete medium over 7 days (for mouse cells) or by the selection of td-tomato-positive cells (for human cells) by FACS. Integrin β3 or ferroportin-1 OE was validated by Western blotting.

### 2.4. In Vitro Proliferation and IC50 Determination 

Cell proliferation was measured using a sulforhodamine B (SRB) colourimetric assay as described previously [23,42] with minor modifications. Briefly, TBCP-1 or TBCP-1NR (1 × 10^3^) cells were seeded in triplicate wells of a 96-well plate in 200 μL of serum-containing medium and their proliferation was compared over 5 days at the indicated time points. Half maximal inhibitory concentrations (IC50) values of various mono- or combination therapies were determined in the same assay over 3 days with an initial cell density of 2 × 10^3^ (TBCP-1 or TBCP-1NR) or 5 × 10^3^ (SKBR3, SKBR3NR, BT474, BT474NR, MCF7-HER2Br3, JIMT1Br3 or SUM190Br) cells/200 μL/well and IC50 values calculated using Hill’s equation in the GraphPad Prism 6.0 software. The nature of the interaction of inhibitor combinations was determined using the Bliss dose–response surface model [43]. Bliss scores, defined as % synergistic cytotoxicity, were calculated by subtracting the observed cytotoxicity from the predicted cytotoxicity (assuming all the interactions are additive) and interactions with Bliss score > 0 were determined to be synergistic. The template for Bliss score determination was kindly provided by Dr. Kym Lowes, Walter and Eliza Hall Institute of Medical Research. 

### 2.5. In Vitro Colony-Formation Assay 

Cell survival at low density was assessed by colony-formation assays, as previously described [42], with minor modifications. A single-cell suspension of TBCP-1 or TBCP-NR cells (500 cells/well/2 mL of complete DMEM) was seeded in 6-well plates in triplicate wells per cell line and colony formation was assessed after 7 days. Cell colonies were stained with a 0.1% (*w*/*v*) crystal violet solution (dissolved in 1:1 methanol/water) for 30 min at RT and the plates were scanned using the Epson Perfection 4870 Photo Scanner. Colonies containing more than 50 cells were counted. The data are shown as colony formation efficiency for each cell line (# of colonies formed/initial seeding density × 100) ± SD of triplicate wells. 

### 2.6. In Vitro Migration and Adhesion Assays 

Haptotactic migration toward selected ECM proteins was determined by transwell migration assay as previously described [32] with minor modifications. Briefly, TBCP-1 or TBCP-1NR cells (2 × 10^5^/100 µL in serum-free DMEM supplemented with 0.05% (*w*/*v*) BSA) were seeded in the upper chamber of triplicate transwells and 600 μL of the same medium was added to the bottom well. Cells were allowed to migrate to the underside of the porous membrane (pre-coated with appropriate ECM) over a 4 h incubation period at 37 °C under 5% CO_2_ and a humidified atmosphere. Three fields per membrane were imaged using the pre-set DAPI filter on a Zeiss Axio Observer Inverted Microscope with a 20× objective lens and the number of migrated cells per field of view was determined by manual counting. The results show a representative experiment and are expressed as the mean number of migrated cells per field of view ± SD of nine fields of view (3 fields of view per membrane × 3 replicate membranes per test condition). 

Cell adhesion to various ECM proteins was determined using a short-term adhesion assay as previously described [32] with minor modifications. Briefly, ECM proteins were diluted to the desired concentration in PBS and 50 µL was added into triplicate wells of a clear-bottom black 96-well tissue culture plate for each condition and incubated overnight at 4 °C. Coated wells were washed once with PBS and blocked with 100 µL/well of a solution of 1% (*w*/*v*) BSA diluted in PBS for 1 h at 37 °C. TBCP-1 or TBCP-1NR cells (1 × 10^6^ cells/mL) were resuspended in serum-free media (SFM) and labelled with 5 µL (2.5 µg) of 1 mg/mL calcein AM solution (Invitrogen, #815297) for 30 min at 37 °C in the dark with occasional shaking. Labelled cells were washed twice with PBS, resuspended at 2 × 10^6^ cells/mL of SFM and added to coated wells in triplicates (100 µL/well). The plates were spun at 1200 rpm for 2 min at 4 °C and incubated at 37 °C for 30 min. Unbound cells were removed by washing the plates thrice with PBS. The remaining adherent cells were lysed using 100 µL/well of 1% (*w*/*v*) SDS. An additional 1 mL of the original labelled cell suspension was used to generate a standard curve. Briefly, 0 µL, 25 µL, 50 µL, 75 µL, and 100 µL of lysed calcein-labelled cells were distributed to uncoated wells in triplicates and volumes were completed to 100 µL with the 1% SDS solution. Fluorescence at 530 nm was measured using a microplate reader (ENSIGHT, PerkinElmer). The % of adhesion in sample wells was extrapolated from the standard curve and plotted using GraphPad Prism 6.0 software. 

### 2.7. Flow Cytometry 

Cell surface expression of various integrin receptor subunits or SLC3A2/CD98hc was measured by standard flow cytometry as previously described [32]. Briefly, the cells (1 × 10^6^) were resuspended in the blocking buffer (DMEM supplemented with 2% BSA and 2% FCS) for 30 min on ice. The cells were then incubated with appropriate primary antibody: Chemicon (anti-integrin β1 MAB 1997 (clone MB1.2); anti-integrin α5 5H10-27 (clone MFR-5); anti-integrin α6 MAB 1378 (clone NKI-GoH3)); BD Pharmingen (anti-integrin β4 553745; anti-integrin αv 552,299 (clone RMV); anti-integrin β3 553343; anti-CD98hc H202-141); Invitrogen (anti-integrin β5 14-0497-82 (clone KN52); anti-integrin α2 14-5971-85 (clone DX5)) or matched isotype control diluted in labelling buffer (DMEM supplemented with 2% FCS) for 1 h on ice. Unbound antibodies were removed by washing twice with PBS, 2% FCS, and the cells were treated with an appropriate fluorescein isothiocyanate (FITC)-conjugated secondary antibody in a labelling buffer for 45 min on ice. The cells were washed again as above and stained with the viability dye 4′, 6′- diamidino-2-phenylindole (DAPI at 0.5 µg/mL) immediately prior to analysis on a BD FACS Canto II Flow Cytometer (BD Biosciences). 

### 2.8. Western Blot

The expression of integrin β3, iron metabolism effectors (transferrin receptor, ferritin, and ferroportin-1), or GPX4 in whole-cell lysates was detected using standard immunoblotting. Primary antibody against integrin β3 (ET1606-49, HuaBio, 1/1000 dilution), ferritin (ab75973, Abcam, 1/2000 dilution), transferrin receptor-1 (TFR-12-M, Alpha Diagnostics, San Antonio, TX, USA, 1/1000 dilution), ferroportin-1 (NBP1-21502, Novus Biologicals, 1 µg/mL), GPX4 (ab125066, Abcam, 1/1000 dilution), and the appropriate horseradish peroxidase (HRP)-conjugated secondary antibody were used to detect the respective proteins. An anti-GAPDH antibody (Abcam ab8245, 0.2 μg/mL) was used as a loading control. 

To analyse the expression of the EGFR family of receptors and downstream signalling effectors, subconfluent cultures were serum-starved overnight in SFM prior to exposure to inhibitors as described previously [23]. Primary antibodies against EGFR (E235, Abcam, ab32077, 1/1000 dilution), phospho-EGFR (Y1173, Abcam ab5652, 1/1000 dilution), HER2 (ab2428, Abcam, 1/200 dilution), phospho-HER2 (Tyr877, Cell Signalling Technology, #2241, 1/1000 dilution), MAPK (ERK1/2) (L34F12, Cell Signalling Technology, #4696, 1/1000 dilution), phospho-MAPK (p-ERK1/2) (Thr 202/Tyr204, Cell Signalling Technology, #9101, 1/1000 dilution), AKT (40D4, Cell Signalling Technology, #2920, 1/1000 dilution), and phospho-AKT (Ser 473, Cell Signalling Technology, #9271, 1/1000 dilution) were used to detect protein targets and specific binding detected using appropriate HRP-conjugated secondary antibodies and enhanced chemiluminescence (ECL) reagents (Amersham Biosciences, Castle Hill, NSW, Australia).

### 2.9. Phalloidin Staining 

Cellular morphology and alterations in actin cytoskeleton in neratinib sensitive and resistant cells or in cells with altered integrin β3 expression were visualised by phalloidin staining as described previously [44], with minor modifications. Briefly, 1 × 10^5^ cells/500 µL well were seeded in multi-chambered glass slides (Lab-Tek II Chamber Slide, Thermo Fischer Scientific, #154534) in DMEM supplemented with 10% FBS and 1% P/S and grown at 37 °C until subconfluent. For weakly adherent human BT474 and BT474NR cells, the chamber slides were pre-coated with 100 µL/well of poly-L-lysine (Sigma, #P4707) to aid attachment. Subsequently, the cells were fixed with pre-warmed 4% (*w*/*v*) paraformaldehyde (PFA) for 10 min at RT and gently washed 3 times with PBS. Fixed cells were permeabilised with 0.1% Triton X-100 for 5 min at RT and stained with 100 µL/well of Alexa-488 conjugated phalloidin solution (1/500 dilution, Abnova, #U0281) containing 0.5 µg/mL DAPI to visualise the nuclei for 90 min at RT in the dark on a rocker. Stained cells were washed gently 3 times with PBS and slides were mounted using the Vectashield Antifade Mounting Medium (Vectorlabs, #H-1000). Fluorescent and brightfield images were taken with a Zeiss Axio Observer Inverted Microscope using the pre-set eGFP, DAPI and brightfield filters.

### 2.10. Cystine Uptake Measurement 

Briefly, 30,000 cells/100 μL/well were plated in a black 96-well plate and grown to confluence. These cells were washed with PBS and incubated with 100 μL/well of pre-warmed Kreb’s Buffer [115 mM NaCl, 2 mM KCl, 1 mM MgCl_2_, 25 mM NaHCO_3_, 0.25% BSA] supplemented with 4.5 g/L D-glucose for 1 h at 37 °C. Subsequently, 50 μL of cystine-FITC diluted in Kreb’s buffer (concentrations ranging from 10 μM to 1000 μM) with or without 100 μM neratinib were added to the wells and incubated for 5 min at 37 °C. After the incubation, the supernatant was removed, and the cells were washed twice with PBS prior to lysis with 100 μL of 1% SDS/well and incubated in the dark at RT on a shaker for 5 min. The fluorescence of an aliquot (2 μL) of each cystine-FITC solution was measured to calculate the amount of cystine-FITC (in μmoles) accumulated in cells. The protein amount per well was quantitated using a standard BCA protein quantitation assay to normalise the data. The data show cystine-FITC uptake rate expressed as nmoles/min.mg of proteins ± SD from 5 replicates.

### 2.11. Glutathione Measurement

The monochlorobimane fluorometric method was used to measure the amount of reduced glutathione (GSH) in cells [45]. Briefly, cells were grown to confluence in 96-well plates and proteins were extracted in 75 µL of 1% digitonin in 50 mM Tris Assay Buffer (pH 7.4). Samples (50 µL) were mixed with 50 µL of Working Solution (100 µM mCB and 1 U/mL glutathione-S-transferase in 50 mM Tris Assay buffer [pH 7.4]) in a black 96-well plate with a clear bottom. The assay plate was incubated in the dark for 60 min at RT. Fluorescence generated by the GSH-mCB adduct was measured at 485 nm using a microplate reader (ENSIGHT, PerkinElmer) with an excitation wavelength of 380 nm. The GSH content (µM) in samples was extrapolated from a standard curve (0–100 µM of reduced GSH diluted in 50 mM Tris Assay buffer [pH 7.4]) and normalised relative to the total protein content in each well. Data show mean ± SD from *n* = 3 experiments, each performed in duplicates.

### 2.12. Tumour Growth Assays and Neratinib Therapy

All procedures involving mice conformed to the National Health and Medical Research Council (NHMRC) animal ethics guidelines and were approved by the Austin Health Animal Ethics Committee (Ethics # A2016/05346 and A2019/05601). Female BALB/C mice were obtained from the Walter and Eliza Hall Institute (WEHI, Melbourne, Australia). Mice were housed in a pathogen-free environment with food and water freely available. Mice were monitored in accordance with the ethics guidelines for signs of ill-health or tumour-associated distress.

Tumour growth assays and neratinib efficacy evaluation were done as previously described [23], with minor modifications. Briefly, female BALB/C mice (6–8 weeks old) were anaesthetized with isoflurane (2% Isoflurane, 2 L/minute O_2_) and 10^6^ viable tumour cells (TBCP-1NR, integrin β3 KO-1, integrin β3 KO-2 or KO-1 + integrin β3 OE) were injected into the 4th inguinal mammary fat pad in 20 μL PBS. To assess the impact of neratinib treatment on primary tumour growth, mice-bearing measurable tumours (approx. 100 mm^3^) were treated once daily with either vehicle control [(0.5% (*w*/*v*) methylcellulose, 0.4% (*v*/*v*) Tween-80] or neratinib (60 mg/kg) by oral gavage until the primary tumours of vehicle control-treated mice reached 1500 mm^3^ or earlier if signs of distress or ulceration at the tumour site was observed. Tumour volumes were measured for the duration of the treatment and primary tumour weights were recorded at the endpoint. 

### 2.13. Histology and Immunohistochemistry 

Tissues were fixed in 10% buffered formalin for 24 h and processed for paraffin embedding. Sections (4 μm) were cut using a Leica RM 2245 Microtome. Expression of integrin β3 was assessed using standard immunohistochemistry as previously described [23] with minor modifications. Briefly, paraffin sections were dewaxed and incubated in antigen retrieval buffer (10 mM citrate buffer pH = 6.0) for 3 min in a pressure cooker at 80 kPa. Sections were stained with anti-integrin β3 antibody (HuaBio ET1606-49) or appropriate isotype overnight at 4 °C. The following day, sections were washed thrice with wash buffer (PBS containing 0.1% Tween-20) and incubated with the appropriate biotin-conjugated secondary antibody for 30 min at RT. The avidin/biotinylated HRP complex (Vectastain ABC reagent, #ZEO717, Vector Laboratories) was added and visualised using the 3,3′-diaminobenzidine chromogen substrate kit (DAKO, K3468). The reaction was stopped with tap water before the development of non-specific staining in the matched isotype control. Sections were counterstained using haematoxylin and mounted in Entellan mounting medium. Brightfield images were captured using a Zeiss Axio Observer Inverted Microscope. Quantitative scoring of IHC staining was done on scanned whole tumour sections using Aperio ImageScope software v11.1.2.760. The expression of integrin β3 was compared across TBCP-1 and TBCP-1NR tumours and scored for the number and intensity of strong positive pixels per unit area. 

### 2.14. Statistical Methods 

All statistical analyses were performed in Graphpad Prism 6 software. Statistical tests used to calculate *p*-values are indicated in the figure legends. Values were considered statistically significant when *p* < 0.05. Unless otherwise indicated, data from in vitro experiments are presented as mean ± SD and data from in vivo experiments are presented as mean ± SEM.

## 3. Results 

### 3.1. Development and Characterisation of Neratinib-Resistant Models of HER2-Positive Breast Cancer 

Neratinib-resistant (NR) mouse and human HER2-positive breast cancer cells were developed by continuous culture in escalating doses of neratinib (up to 2 μM) over several months. These resistant models display >10-fold decrease in sensitivity to neratinib compared to age-matched sensitive parental cells (PAR) cultured in the absence of neratinib (Table 1). Interestingly, resistant cells also acquired cross-resistance to other HER2-targeting TKIs that do not directly promote ferroptosis (lapatinib and tucatinib) [23]. Notably, resistant cells also showed reduced sensitivity to classical ferroptosis inducers (Erastin and RSL3) (Table 1) indicating a close relationship between resistance to ferroptosis and resistance to TKIs more broadly. 

**Figure 1:**
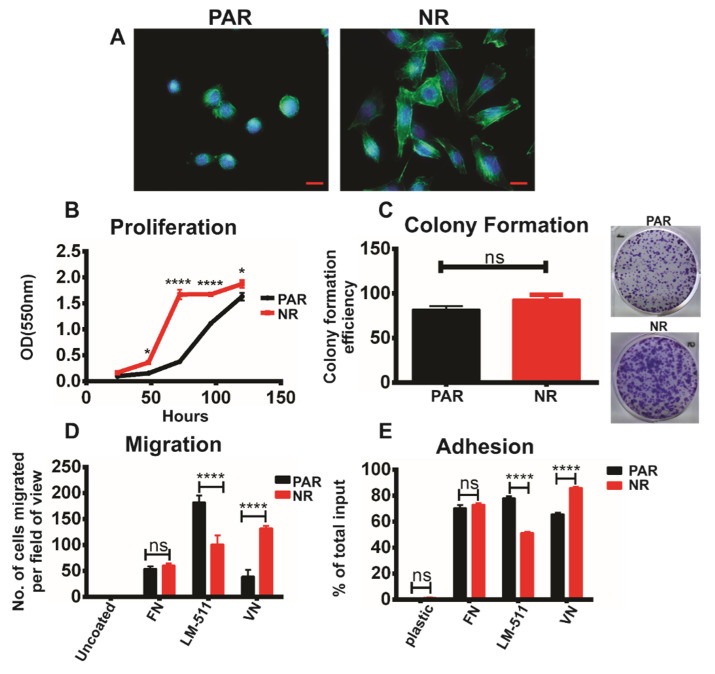
Morphological and functional characterisation of TKI-resistant cells in vitro. (**A**) The alterations in morphology and actin cytoskeleton were visualised in the resistant TBCP-1NR cells (NR) and compared to age-matched sensitive TBCP-1 cells (PAR) by F-actin staining using A488-conjugated phalloidin. Red (Scale bar) = 50 μm. Blue (DAPI) = nuclei. Green (A488-phalloidin) = F-actin. (**B**) Cell proliferation was measured in TBCP-1 (PAR) or TBCP-1NR (NR) cells using a sulforhodamine B (SRB) colourimetric assay. Cells were seeded at 1000 cells/well in triplicates in 96-well plates and cultured over 5 days. Cell proliferation was measured at the indicated time points. Each point on the curve represents the mean ± SD of triplicates of the respective time point from a representative experiment of three independent experiments (*n* = 3). Statistical significance was determined using two-way ANOVA, Bonferroni’s multiple comparison test; *p* < 0.05 was considered significant, * *p* < 0.05, **** *p* < 0.0001. (**C**) Colony formation was measured in TBCP-1 (PAR) or TBCP-1NR (NR) cells over 10 days. Cells were seeded at 500 cells/well in a 6-well plate in triplicates and colonies were fixed with methanol and stained with crystal violet. Data show mean colony-formation efficiency (no. of colonies formed/total no. of cells plated × 100) ± SD of triplicates from a representative experiment of three independent experiments (*n* = 3). Statistical significance was determined using the Mann–Whitney *t*-test; *p* < 0.05 was considered significant, ns = not significant. (**D**) Haptotactic migration of TBCP-1 (PAR) or TBCP-1NR (NR) cells in response to the indicated ECM proteins was evaluated in Transwell chambers and the number of migrating cells per field of view was determined manually by counting DAPI-positive cells on the underside of the porous membrane. The data show mean ± SD of nine replicates (3 fields of view per membrane × triplicate membranes per condition) from a representative experiment of three independent experiments (*n* = 3). (**E**) The ability of TBCP-1 (PAR) or TBCP-1NR (NR) cells to adhere to the indicated ECM proteins was determined in a short-term (30 min) assay and data are shown as mean ± SD of triplicates from a representative experiment of three independent (*n* = 3) experiments. Statistical significance for migration and adhesion assays was determined using two-way ANOVA, Tukey’s multiple comparison test; *p* < 0.05 was considered significant, **** *p* < 0.0001, ns = not significant. VN = vitronectin, LM-511 = laminin-511, and FN = fibronectin.

Interestingly, TKI-resistant cell lines developed an elongated/spread morphology with prominent actin stress fibres, characteristic of motile mesenchymal cells, a trait commonly associated with drug resistance [46]. In contrast, age-matched parental (TKI sensitive) cells maintained an epithelial-like morphology (Figure 1A and Supplementary Figure S1A). We further sought to determine changes in functional properties that may be associated with the remodelling of the actin cytoskeleton and the acquisition of resistance to TKI-induced ferroptosis. For this, we compared the proliferative, adhesive, and migratory properties of TBCP-1 and TBCP-1-NR cells in vitro. Resistant cells showed an increased proliferation rate over 5 days (Figure 1B). Of note, the cells were also counted at each time point to confirm that the increased absorbance values in the SRB colourimetric assay were not due to differences in morphology and SRB dye uptake but rather than an increase in cell number. In contrast, there was no difference in the number of colonies formed by sensitive or resistant cells when seeded at low density. However, resistant cells tended to form larger colonies when plated at low density (Figure 1C). Collectively these results indicate increased intrinsic proliferation but not viability in TKI-resistant cells in standard cultures. Migration and adhesion to vitronectin (VN) increased in resistant cells whereas these responses to laminin-511 (LM-511) were decreased compared to TKI-sensitive parental cells (Figure 1D,E). Adhesion to and migration towards fibronectin were unchanged. 

### 3.2. TKI Resistance Is Associated with Increased Expression of αvβ3 Integrin

**Figure 2:**
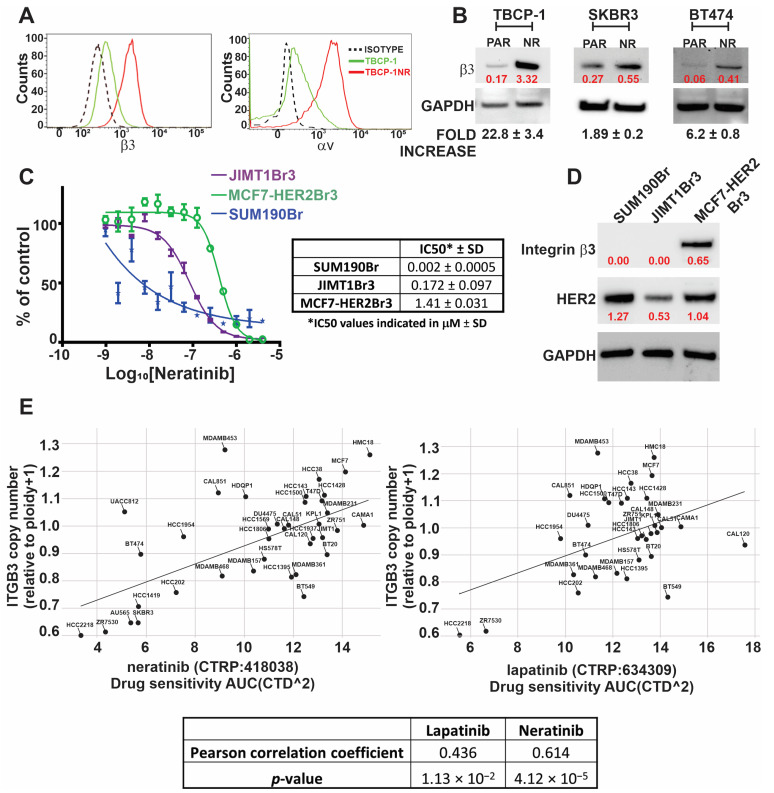
TKI resistance is associated with increased expression of αvβ3 integrin. (**A**) Cell surface expression of the αv and β3 integrin subunits was analysed in TBCP-1 or TBCP-1NR cells by flow cytometry. Black dashed line = isotype control, green = TBCP-1 and red = TBCP-1NR. (**B**) Expression levels of integrin β3 were compared in mouse or human TKI-sensitive and resistant pairs by Western blot analysis of whole cell lysates and were normalised relative to GAPDH. The data show representative blots and mean fold increase relative to matched sensitive pairs ± SD from three independent lysates (*n* = 3). The intensity ratio of each band relative to GAPDH is indicated in red. (**C**) Sensitivity of the indicated human brain metastatic HER2-positive breast cancer cell lines to neratinib was determined in a 3-day SRB colourimetric assay. The data show a representative curve and mean IC50 values ± SD for each cell line from three independent experiments (*n* = 3). (**D**) Western blot analysis of integrin β3 and HER2 expression in whole cell lysates of human brain metastatic lines. GAPDH was used as the loading control. The intensity ratio of each band relative to GAPDH is indicated in red. (**E**) The association between TKI resistance and integrin β3 expression in breast cancer cell models was analysed using Cancer Dependency Map (DepMap) analysis tool. The data show the association of ITGB3 copy number (log2 relative to ploidy +1, in 21Q1 dataset) with neratinib (AUC values from CTRP: 418,038 dataset) or lapatinib sensitivity (AUC values from CTRP:634309 dataset) for a panel of 38 breast cancer cell lines. The strength of the linear association was determined by measurement of Pearson coefficient and linear regression t-test was used to determine the slope of the regression and statistical significance, *p* < 0.05 was considered significant. The uncropped blots are shown in Supplementary File S1.

Based on the above observations, we hypothesised that TKI resistance might be associated with changes in the expression of integrin receptors, important mediators of cellular interaction with the tumour matrix microenvironment [47]. Cell surface expression of multiple integrin receptors was analysed by flow cytometry. Notably, there was a significant increase in the expression of both subunits of the VN receptor, αvβ3 integrin, in TBCP-1NR compared to TBCP-1 cells (Figure 2A). The expression of other integrins was either unchanged (β1, β5, α2, and α6) or reduced slightly (β4 and β5) (Supplementary Figure S1B). Importantly, the expression of integrin β3 was consistently increased across three batches of TBCP-1NR cells derived independently by long-term culture in the presence of increasing concentrations of neratinib (Supplementary Figure S1C). Increased expression of integrin β3 was also confirmed by Western blotting of whole cell lysates from TBCP-1NR or human TKI-resistant SKBR3NR and BT474NR cells compared to their respective age-matched TKI-sensitive parental cells (Figure 2B). The correlative association between integrin β3 expression and neratinib resistance was further examined in three human brain-metastatic HER2-positive breast cancer cell lines. Notably, HER2^high^/β3^high^ MCF7-HER2Br3 cells were highly resistant to neratinib whereas HER2^high^ SUM 190Br cells that lack integrin β3 were highly sensitive to neratinib. JIMT1Br3 cells showed moderate expression of HER2, lack of β3 integrin expression, and moderate response to neratinib (Figure 2C,D). To further investigate if integrin β3 is predictive of TKI sensitivity in other human breast cancer models, we analysed the association between integrin β3 gene copy number and lapatinib or neratinib sensitivity in multiple human breast cancer cell lines using the Cancer Dependency Map (DepMap) Analysis tool [48,49]. For these analyses, we interrogated the association between ITGB3 gene copy number (21Q1 dataset) and neratinib sensitivity (AUC values from CTRP: 418,038 dataset) or lapatinib sensitivity (AUC values from CTRP:634309 dataset). AUC values for a drug refers to the “area under the curve” for a dose–response curve. Lower AUC reflects the higher sensitivity of a cell line to the drug of interest [50]. Importantly, a comparison of 38 cancer cell lines found a highly significant correlation between high integrin β3 gene copy number and neratinib (Pearson correlation coefficients: 0.614, *p*-value = 4.12 × 10^−5^) or lapatinib (Pearson correlation coefficients: 0.436, *p*-value = 1.13 × 10^−2^) resistance (Figure 2E). These trends were also maintained when only HER2-positive cell lines were selected but did not reach statistical significance due to the limited number of data points. Taken together, these results illustrate the strong correlation between high integrin β3 expression and TKI resistance.

### 3.3. αvβ3 Integrin Functionally Regulates Resistance to TKI-Induced Ferroptosis 

**Figure 3:**
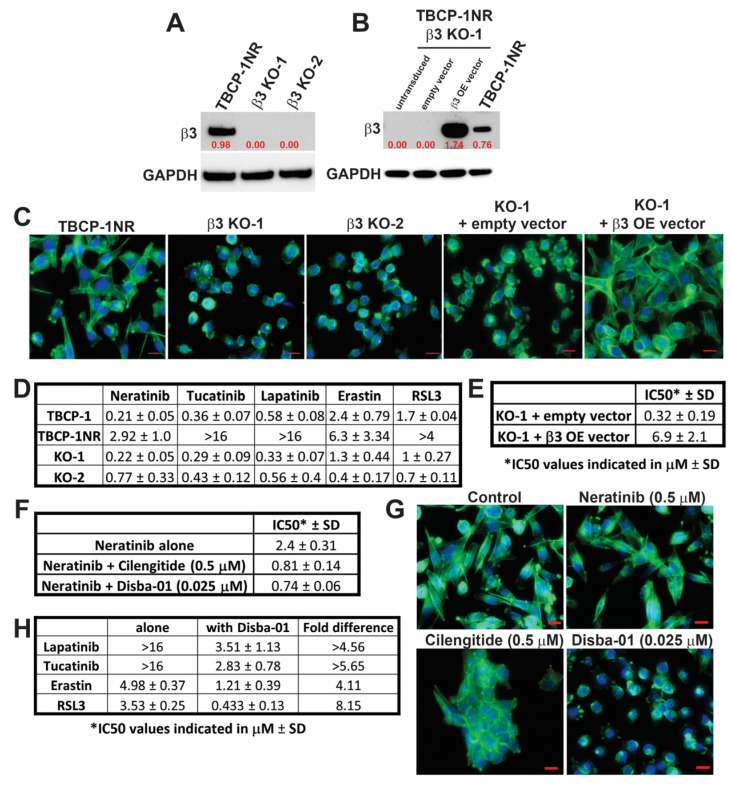
Integrin β3 functionally regulates morphology and TKI response in HER2-positive cells in vitro. (**A**) Genetic KO of integrin β3 was confirmed in the indicated TBCP-1NR clones (KO-1 and KO-2) by Western blot analysis of whole-cell lysates and GAPDH was used as loading control. The intensity ratio of each band relative to GAPDH is indicated in red. (**B**) Overexpression of integrin β3 in TBCP-1NR β3 KO-1 cells relative to untransduced or empty vector transduced cells was validated by Western blot analysis of whole cell lysates and GAPDH was used as loading control. The intensity ratio of each band relative to GAPDH is indicated in red. (**C**) Morphological changes in integrin β3 control, KO or OE cells were visualised by F-actin staining using A488-conjugated phalloidin. Red (Scale bar) = 50 μm. Blue (DAPI) = nuclei and Green (A488-phalloidin) = F-actin. (**D**) The sensitivity of integrin β3 KO clones to TKIs (neratinib, lapatinib, and tucatinib) and ferroptosis inducers (Erastin and RSL3) was determined using a 3-day SRB assay. The data show mean IC50 ± SD from three independent experiments (*n* = 3). (**E**) Sensitivity of empty vector, or integrin β3 OE vector transduced β3 KO-1 cells to neratinib was determined in a 3-day SRB assay. The data show mean IC50 ± SD from three independent experiments (*n* = 3). (**F**) The potency of αvβ3 integrin inhibitors (Cilengitide or Disba-01) was assessed in combination with neratinib in a 3-day SRB assay. The data show mean IC50 ± SD from three independent experiments (*n* = 3). (**G**) Morphological changes following neratinib (0.5 μM), Cilengitide (0.5 μM) or Disba-01 (0.025 μM) treatment were visualised by F-actin staining using A488-conjugated phalloidin. Red (Scale bar) = 50 μm. Blue (DAPI) = nuclei and Green (A488-phalloidin) = F-actin. (**H**) The potency of Disba-01 (0.025 μM) in combination with lapatinib, tucatinib, Erastin or RSL3 was assessed using a 3-day SRB assay. The data show mean IC50 ± SD from three independent experiments (*n* = 3). The uncropped blots are shown in Supplementary File S1.

To investigate if integrin β3 contributes functionally to TKI and ferroptosis resistance, its expression was deleted in TBCP-1NR cells using CRISPR/Cas9 gene-editing technology. KO of β3 integrin protein was confirmed in two clones (KO-1 and KO-2) by Western blot analysis (Figure 3A). Moreover, TBCP-1NR β3 KO-1 cells were transduced with an empty vector (Control) or integrin β3 OE lentiviral vector and integrin β3 expression status of the puromycin-resistant cells was validated by Western blot (Figure 3B). Interestingly, genetic KO of integrin β3 reversed the mesenchymal-like morphology of TBCP-1NR cells while forced OE of the protein in the KO-1 cells re-instated this phenotype (Figure 3C). As expected and consistent with the coordinated expression of αv and β3 integrin subunits reported in other tumour models [31], integrin β3 KO clones showed a concomitant decrease in the expression of αv subunit (Supplementary Figure S2A). No consistent changes were observed in the expression of other integrin subunits analysed. Moreover, forced OE of integrin β3 in TBCP-1NR integrin β3 KO-1 cells restored high αv integrin expression (Supplementary Figure S2B). 

Importantly, integrin β3 deletion in TBCP-1NR cells reversed resistance to neratinib in vitro (Figure 3D). Notably, neratinib-induced cell death in these clones was prevented in the presence of the ferroptosis inhibitor, liproxstatin-1 (Supplementary Figure S2C), as observed in neratinib sensitive TBCP-1 parental cells [23]. This indicates that integrin β3 KO is sufficient to re-sensitise TBCP-1NR cells to neratinib-induced ferroptosis. In addition, integrin β3 KO cells were re-sensitised to other HER2-targeting non-ferroptosis inducing TKIs or to ferroptosis inducers (Figure 3D), further demonstrating that the effect of integrin β3 receptor KO on therapy response is not restricted to neratinib and may even extend to other non-ferroptosis-inducing therapies. Moreover, neratinib resistance was re-induced upon integrin β3 OE in the KO-1 cells (Figure 3E). To further validate these observations in human HER2-positive breast cancer cells, integrin β3 KO MCF7-HER2Br3 cells were generated using CRISPR/Cas9 gene editing. Two single-cell clones were confirmed for the loss of integrin β3 protein expression by Western blot analysis (Supplementary Figure S3A). Conversely, integrin β3 was overexpressed in the β3^low^ SKBR3 and BT474 human HER2-positive cell lines (Supplementary Figure S3B,C). While integrin β3 KO did not significantly alter the morphology of MCF7-HER2Br3 cells, both KO clones regained sensitivity to neratinib compared to parental cells (Supplementary Figure S3E,F). Importantly, integrin β3-overexpressing BT474 and SKBR3 cells adopted an elongated mesenchymal-like morphology (Supplementary Figure S3D) and their sensitivity to neratinib decreased by 6- and 9-fold, respectively (Supplementary Figure S3E,F). Collectively, these findings unequivocally demonstrate the pivotal function of αvβ3 integrin in mediating resistance to neratinib-induced ferroptosis and to other HER2-targeting TKIs or ferroptosis inducers. 

Next, we asked whether pharmacological inhibition of this receptor could be an effective strategy to reverse resistance to TKI-induced ferroptosis. Two αvβ3 integrin inhibitors were selected: Cilengitide, a potent cyclic arginine-glycine-aspartic acid (RGD)-containing pentapeptide, is one of the best-characterised inhibitors that selectively inhibits αvβ3 and αvβ5 integrins [51,52], and Disba-01, an αvβ3-targeting disintegrin derived from the venom of *Bothrops alternatus* [41]. The effect of αvβ3 integrin inhibition on TBCP-1NR cell proliferation was assessed using a standard SRB assay. While Disba-01 alone partially inhibited proliferation at concentrations above 0.050 µM, Cilengitide used at up to 1 μM had no effect on the proliferation of these cells. To evaluate the impact of these integrin inhibitors on neratinib sensitivity, TBCP-1NR cells were treated with increasing concentrations of neratinib alone or in combination with a suboptimal dose of Disba-01 (0.025µM) or Cilengitide (0.5 µM) that do not inhibit proliferation. Notably, either αvβ3 integrin inhibitor re-sensitised TBCP-1NR cells to neratinib and significantly reduced neratinib’s IC50 (Figure 3F). Interestingly, the Disba-01 + neratinib combination was more potent than the Cilengitide + neratinib combination, as evidenced by a significant reduction in IC50 even at low nanomolar concentrations of Disba-01. We also confirmed that Cilengitide (0.5 µM) or Disba-01 (0.025 µM) alone and in combination with neratinib disrupted F-actin stress fibres formation and reversed the mesenchymal-like morphology of TBCP-1NR cells as observed with integrin β3 receptor KO. Notably, while treatment with Cilengitide induced cell-clumping and formation of aggregates, it only partially disrupted the mesenchymal-like morphology. In contrast, cells treated with Disba-01 fully reverted to a round epithelial-like morphology, without clumping or aggregation (Figure 3G).

We further sought to determine if the synergistic activity of neratinib + αvβ3 integrin inhibitors in resistant TBCP-1NR cells could extend to other TKIs or ferroptosis inducers. For these assays, Disba-01 (0.025 μM) was combined with increasing concentrations of HER2-targeting TKIs (lapatinib or tucatinib) or ferroptosis inducers (Erastin or RSL3). While Disba-01 partially restored sensitivity to lapatinib (IC50 = 3.51 μM, >4.56-fold decrease) and tucatinib (IC50 = 2.83 μM, >5.65-fold decrease), (Figure 3H), it restored the sensitivity of TBCP-1NR cells to Erastin (IC50 = 1.21 μM) and RSL3 (IC50 = 0.433 μM) to the same extent as integrin β3 receptor KO (Figure 3H). To determine the nature of these drug interactions, data from the above assays were analysed using a 3D analytical method called Bliss dose–response surface model [43]. Bliss scores, defined as % synergistic cytotoxicity, were calculated by subtracting the observed cytotoxicity from the predicted cytotoxicity (assuming all the interactions are additive) and interactions with Bliss score > 0 were determined to be synergistic. As shown in the 3D plots (Supplementary Figure S4A,B), either αvβ3 integrin inhibitor showed high synergy in combination with neratinib. However, the dose of Disba-01 required to observe a synergy was 20-fold lower than that of Cilengitide. A similar synergy between Disba-01 and other TKIs and ferroptosis inducers was confirmed using this Bliss dose–response surface model (Supplementary Figure S4C–F). Collectively, these results indicate that pharmacological inhibition of αvβ3 integrin mimics the effect of β3 integrin receptor KO on reversing resistance to neratinib-induced ferroptosis as well as cross-resistance to other ferroptosis inducers (Erastin and RSL3), and partially restores the sensitivity of neratinib-resistant cells to other non-ferroptotic HER2-targeting TKIs (tucatinib and lapatinib). 

### 3.4. αvβ3 Integrin Mediates TKI Resistance through Persistent Activation of AKT Signalling

**Figure 4:**
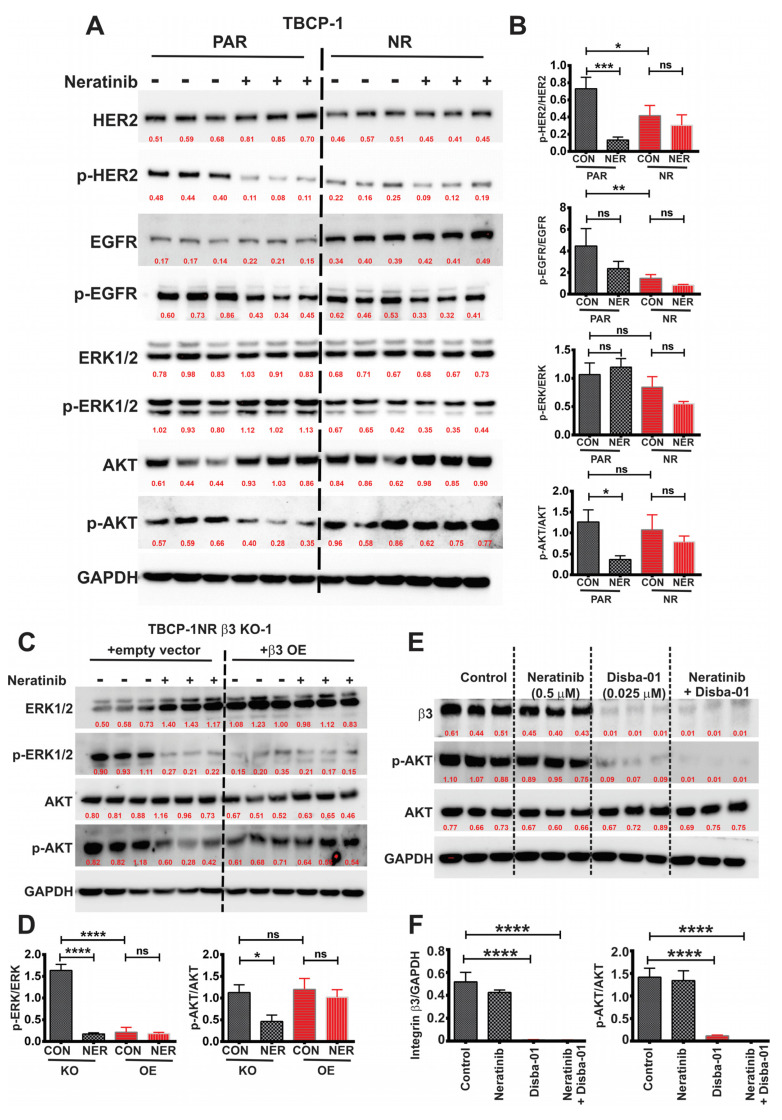
Integrin β3 signals through AKT activation in TKI-resistant cells. (**A**) The impact of neratinib treatment on EGFR-HER2 signalling was analysed in TBCP-1 age-matched sensitive (PAR) and TKI-resistant (NR) cells. Changes in the levels of the indicated signalling intermediates were measured by Western blot in serum-starved cells following 1 h of vehicle control (DMSO) or neratinib (0.5 μM) treatment and 10 min EGF stimulation. GAPDH was used as the loading control. The intensity ratio of each band relative to GAPDH is indicated in red. (**B**) Data shows quantitation of mean phospho/total protein ratio ± SD from three independent experiments (*n* = 3). Statistical significance was determined by one-way ANOVA, Holm–Sidak’s multiple comparison test, *p* < 0.05 was considered significant, * *p* < 0.05, ** *p* < 0.005, *** *p* < 0.001, ns = not significant. (**C**) The impact of neratinib treatment on ERK and AKT phosphorylation was analysed in TBCP-1NR integrin β3 KO cells transduced with empty vector or integrin β3 OE vector. Changes in the levels of the indicated signalling intermediates were measured by Western blot in serum-starved cells following 1 h of vehicle control (DMSO) or neratinib (0.5 μM) treatment and 10 min EGF stimulation. GAPDH was used as the loading control. The intensity ratio of each band relative to GAPDH is indicated in red. (**D**) Data show quantitation of mean phospho/total protein ratio ± SD from three independent experiments (*n* = 3). (**E**) The impact of Disba-01 (0.025 μM) treatment alone or in combination with neratinib (0.5 μM) on the expression of integrin β3 and phosphorylation of AKT was determined in TBCP-1NR cells by Western blotting. Briefly, TBCP-1NR cells were serum-starved, treated with the indicated concentration of inhibitors, and stimulated for 10 min with EGF. GAPDH was used as the loading control. The intensity ratio of each band relative to GAPDH is indicated in red. (**F**) Data show mean integrin β3 levels relative to GAPDH or mean phospho/total AKT ratio ± SD from three independent lysates (*n* = 3). Statistical significance (panels **D** and **F**) was determined by one-way ANOVA, Holm–Sidak’s multiple comparison test, *p* < 0.05 was considered significant, **** *p* < 0.0001. The uncropped blots are shown in Supplementary File S1.

We sought to understand the precise mechanisms by which αvβ3 integrin mediates resistance to TKI-induced ferroptosis. Given that neratinib primarily targets EGFR/HER2, we first compared the differential signalling response of sensitive and TKI-resistant cells to neratinib treatment, focussing on the EGFR/HER2 downstream signalling. TBCP-1 age-matched sensitive (PAR) and resistant cells (NR) were serum-starved, pre-treated with vehicle control (DMSO) or neratinib (0.5 μM, 1 h), and then stimulated with EGF (100 ng/mL) for 10 min. As expected, neratinib inhibited HER2 (and to a lesser extent, EGFR, *p* > 0.05) phosphorylation in parental TBCP-1 cells. Interestingly, the total HER2 expression, and more significantly HER2 phosphorylation, were lower than in TBCP-1NR cells compared to parental cells whereas the total EGFR expression was elevated. However, in contrast to parental cells, phosphorylation of neither of these receptors was inhibited by neratinib in TBCP-1NR cells (Figure 4A,B). Neratinib failed to inhibit ERK 1/2 phosphorylation in either parental TBCP-1 or TBCP-1NR cells. In contrast, neratinib strongly inhibited AKT activation in parental but not in resistant cells (Figure 4A,B). Similar analyses were done in the human SKBR3 versus SKBR3NR pair. Again, HER2 and AKT phosphorylation were inhibited by neratinib (0.005 μM) in parental SKBR3 cells but not in SKBR3NR cells (Supplementary Figure S5A,B). Total EGFR levels did not change between cell lines, but its phosphorylated form was not detectable under the conditions used. While EGF-induced ERK 1/2 phosphorylation was reduced in SKBR3NR cells compared to parental cells (Supplementary Figure S5A), in agreement with the results obtained in the TBCP-1/TBCP-1NR pair, ERK 1/2 phosphorylation relative to total ERK 1/2 was not inhibited significantly by neratinib (Supplementary Figure S5A,B).

Given the increased expression of αvβ3 integrin in TKI-resistant cells and the widely documented crosstalk between receptor tyrosine kinases (RTKs) and integrins [53,54], we investigated the role of integrin β3 in modulating HER2 downstream signalling in TKI sensitive and resistant lines. For these experiments, TBCP-1NR integrin β3 KO-1 cells transduced with an empty vector or integrin β3 expression vector were serum-starved and pre-treated with neratinib (0.5 μM, 1 h), followed by EGF stimulation (10 min). Under these conditions, ERK1/2 and AKT phosphorylation was inhibited significantly by neratinib in cells lacking integrin β3. Integrin β3-overexpressing cells showed higher expression of ERK 1/2 than control cells and lower EGF-induced ERK 1/2 phosphorylation, but ERK 1/2 phosphorylation was not inhibited further by neratinib (Figure S4C,D). Remarkably, much like the TBCP-1/TBCP-1NR pair, neratinib inhibited EGF-induced AKT phosphorylation in TBCP-1NR β3KO cells lacking integrin β3 but failed to inhibit AKT phosphorylation in the same cells re-expressing integrin β3 (Figure S4C,D). These observations were also replicated in human BT474 cells transduced with an empty vector versus BT474 overexpressing integrin β3 (Supplementary Figure S5C,D). Importantly, treatment with a low dose of Disba-01 (0.025 μM) alone or in combination with neratinib led to the rapid downregulation of integrin β3 receptor expression and a significant reduction in AKT phosphorylation (Figure 4E,F). Taken together, these results indicate a functional association between resistance to neratinib-induced ferroptosis, high integrin β3 expression, and sustained AKT activation. In light of these findings, we asked if direct AKT inhibition would be sufficient to synergistically reverse neratinib resistance. For this, the potent AKT inhibitor (AKT Inhibitor VIII) was first evaluated alone in TBCP-1NR cells. The IC50 for this inhibitor alone was estimated as 1.37 µM (Supplementary Figure S5E). A sub-optimal dose of 1 μM was used in combination with increasing concentrations of neratinib for synergy evaluation (Supplementary Figure S5F). Unlike the synergy observed between Disba-01 and neratinib (Supplementary Figure S4), AKT inhibition failed to restore sensitivity to neratinib (Supplementary Figure S5F). A higher concentration of AKT inhibitor VIII (2 μM) improved response to neratinib in this assay (Supplementary Figure S5F) but AKT inhibition alone was cytotoxic at this concentration (>50% cell death) making it difficult to assess the synergistic nature of AKT inhibitor VIII/neratinib combination. Collectively, these results indicate that pharmacological inhibition of β3 integrin synergistically reverses resistance to TKI-induced ferroptosis more potently than direct AKT inhibition. We speculated that this might be due to the potential involvement of other integrin β3-regulated processes that contribute to resistance. 

### 3.5. αvβ3 Integrin Mediates Resistance to Ferroptosis through Crosstalk with Iron Metabolism and Antioxidant Response Pathways

**Figure 5:**
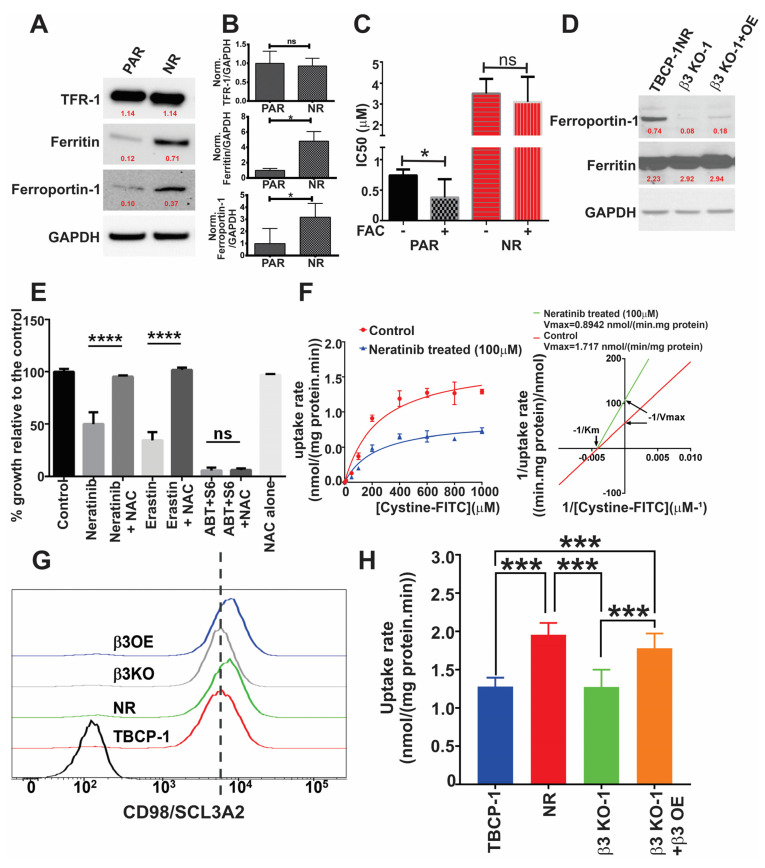
Integrin β3 modulates iron metabolism and antioxidant response in TKI-resistant cells. (**A**) Changes in the basal expression of iron metabolism effectors were analysed in the age-matched sensitive (PAR) or resistant (NR) TBCP-1 cell lysates by Western blot and GAPDH was used as loading control. The intensity ratio of each band relative to GAPDH is indicated in red. The data show representative blots and (**B**) normalised expression relative to GAPDH ± SD from three independent lysates (*n* = 3). Statistical significance was determined by paired *t*-test, *p* < 0.05 was considered significant, * *p* < 0.05, ns = not significant. (**C**) The impact of addition of exogenous iron (500 μM Ferric Ammonium Citrate, FAC) on neratinib sensitivity of TBCP-1 or TBCP-1NR cells was determined using a 3-day SRB assay. Data shows mean IC50 ± SD from three independent experiments (*n* = 3). Statistical significance was determined using a non-parametric Wilcoxon matched-pair *t*-test, ns = not significant, * *p* < 0.05. (**D**) Changes in the basal expression of ferroportin-1 and ferritin were analysed in TBCP-1NR control or integrin β3 KO or OE whole cell lysates by Western blot and GAPDH was used as loading control. The intensity ratio of each band relative to GAPDH is indicated in red. The data show representative blots from two independent experiments (*n* = 2). (**E**) Neratinib (0.3 μM) or Erastin (5 μM)-induced cell death in TBCP-1 cells was prevented by the addition of NAC (2 mM). Cell death induced by the combination of ABT263 (0.5 μM) and S63845 (0.5 μM) was not blocked in combination with NAC. Cells were treated with the indicated inhibitors and growth relative to control was determined after 72 h using a 3-day SRB assay. Data show mean percentage growth relative to control ± SD of triplicates from a representative experiment of three independent experiments (*n* = 3). Statistical significance was determined using two-way ANOVA Tukey’s multiple comparison test. *p* < 0.05 was considered significant. **** *p* < 0.0001 ns = not significant. (**F**) The impact of neratinib treatment on System Xc- activity was determined by quantitation of FITC-labelled cystine uptake (0–1000 μM) in control or neratinib (100 μM)-treated TBCP-1 cells. Data are expressed as uptake rate (nmol/(mg protein.min)) (left panel) or represented as double reciprocal Lineweaver Burk plot (right panel) indicating reduced Vmax but same Km in neratinib treated TBCP-1 cells. (**G**) Cell surface expression of SLC3A2/CD98 was analysed in TBCP-1, TBCP-1NR, integrin β3 KO, and OE cells using standard flow cytometry. Data is presented as a staggered plot to highlight subtle changes in expression profiles. (**H**) TKI resistance and integrin β3 overexpression were characterised by increased uptake of FITC-labelled cystine in mouse TBCP-1 cells. Data are expressed as uptake rate (nmol/(mg protein.min)) and statistical significance was determined using the Mann–Whitney *t*-test, *p* < 0.05 was considered significant, *** *p* < 0.001. The uncropped blots are shown in Supplementary File S1.

Given the well-documented role of iron metabolism effectors in regulating ferroptosis in TBCP-1 cells [23] and other models [14,20,21,22], we analysed the expression of these proteins across sensitive and resistant cells. Compared to parental cells, expression of the iron importer, TFR-1, was unchanged in TBCP-1NR. However, there was a significant increase in the expression of iron storage protein, ferritin, and iron export protein, ferroportin-1, in TBCP-1NR cells compared to age-matched sensitive cells (Figure 5A,B). The expression of ferroportin-1 was also consistently increased in human neratinib-resistant variants compared to age-matched sensitive cells (Supplementary Figure S6A,B). To confirm the functional relevance of increased ferroportin-1 expression, TBCP-1 or TBCP-1-NR cells were treated with increasing concentrations of neratinib, in the presence or absence of 500 μM iron (Ferric Ammonium Citrate, FAC), in a 3-day SRB colourimetric assay. Notably, the sensitivity of parental TBCP-1 cells to neratinib increased significantly (~2-fold, *p* = 0.03) in the presence of supplemental iron. In contrast, the sensitivity of the TBCP-1-NR neratinib-resistant variant was not affected by the addition of iron (*p* = 0.7) (Figure 5C). Interestingly, among the brain-metastatic lines examined, ferroportin-1 expression correlated well with integrin β3 expression and resistance to neratinib. Notably, the highest levels of ferroportin-1 were detected in the integrin β3^high^ neratinib resistant MCF7-HER2Br3 cells (Supplementary Figure S6C). Consistent with this, the deletion of integrin β3 in TBCP-1NR cells decreased the expression of ferroportin-1 but not ferritin (Figure 5D). However, the level of ferroportin-1 was only partially restored by the re-expression of integrin β3 (OE) in the β3 KO cells. 

In light of these observations, we questioned whether the deletion of ferroportin-1 could restore sensitivity to TKI-induced ferroptosis. Ferroportin-1 expression was deleted in MCF7-HER2 Br3 cells using CRISPR/Cas9 technology, and two clones (Fpn KO-1 and Fpn KO-2) were confirmed to lack ferroportin-1 protein expression by Western blot analysis (Supplementary Figure S7A). Surprisingly, neither of the clones showed any difference in sensitivity to neratinib (Supplementary Figure S7B). Furthermore, the overexpression of this receptor in neratinib-sensitive SKBR3 cells did not significantly the alter neratinib response (Supplementary Figure S7C,D). Instead, we observed a significant downregulation of TFR1 and upregulation of ferritin in ferroportin-1 KO cells (Supplementary Figure S7E). Thus, while TKI resistance and elevated integrin β3 expression are characterised by an enhanced expression of ferroportin-1 and higher iron tolerance, genetic ablation of ferroportin-1 is insufficient alone to restore TKI sensitivity. 

Next, we investigated changes in the redox machinery in TKI-resistant cells. We analysed the expression of the central detoxifying enzyme GPX4, previously shown to protect against ferroptosis, and implicated in resistance to TKIs [26,55]. However, no significant differences in GPX4 expression were observed between mouse or human TKI-sensitive versus their resistant variants (Supplementary Figure S6D). Interestingly, we found that N-acetyl cystine (NAC) prevented neratinib-induced ferroptosis but not apoptosis induced by BH3 mimetics in sensitive TBCP-1 cells (Figure 5E). NAC also prevented ferroptosis induced by Erastin, a known inhibitor of the cystine-glutamate antiporter (System Xc-). Hence, we hypothesised that neratinib activity may be mediated in part through inhibition of System Xc-. Indeed, neratinib inhibited the activity of System Xc- non-competitively (FITC-labelled cystine uptake) in mouse TBCP-1 cells. This is evidenced by the reduced Vmax (maximum rate of cystine uptake) but similar Km (System Xc-’s affinity for its substrate) (Figure 5F). Neratinib treatment also inhibited cystine uptake in human SKBR3 and BT474 cells (Supplementary Figure S6E,F). In contrast and in agreement with previous reports showing that lapatinib alone does not promote ferroptosis [23,24], lapatinib treatment, unlike neratinib treatment, was insufficient to inhibit System Xc- in TBCP-1 cells (Supplementary Figure S6G). These findings indicate that the blockade of cystine import is an important feature of neratinib-induced ferroptosis.

Consistent with these observations, TBCP-1NR cells showed a small but reproducible increase in the expression of SLC3A2/CD98 (the regulatory subunit of System Xc-) (Figure 5G). SCL3A2 is known to interact with and modulate integrin signalling [56,57], suggesting a potential link with elevated β3 integrin in neratinib-resistant cells. Importantly, we found that integrin β3 KO was associated with a concomitant reduction in the expression of SLC3A2 while β3 OE restored its expression to the level seen in TBCP-1NR cells (Figure 5G). Furthermore, System Xc- activity correlated with TKI resistance and integrin β3 expression in mouse TBCP-1 and human BT474 models, as evidenced by increased cystine-FITC uptake in TKI resistant or β3 integrin-overexpressing variants and decreased uptake in β3 integrin KO variants (Figure 5H and Supplementary Figure S6H). In addition, short-term treatment with neratinib reduced the level of intracellular antioxidant GSH in parental TBCP-1 cells whereas neratinib-resistant TBCP-1NR cells showed a higher basal level of GSH that was not significantly decreased by neratinib treatment (Supplementary Figure S6I). Collectively, these observations indicate that neratinib promotes ferroptosis in part by inhibiting the activity of System Xc- and that resistance to neratinib-induced ferroptosis is mediated through a β3 integrin-dependent reprogramming of iron and antioxidant metabolism.

### 3.6. αvβ3 Integrin Mediates Neratinib Resistance In Vivo 

**Figure 6:**
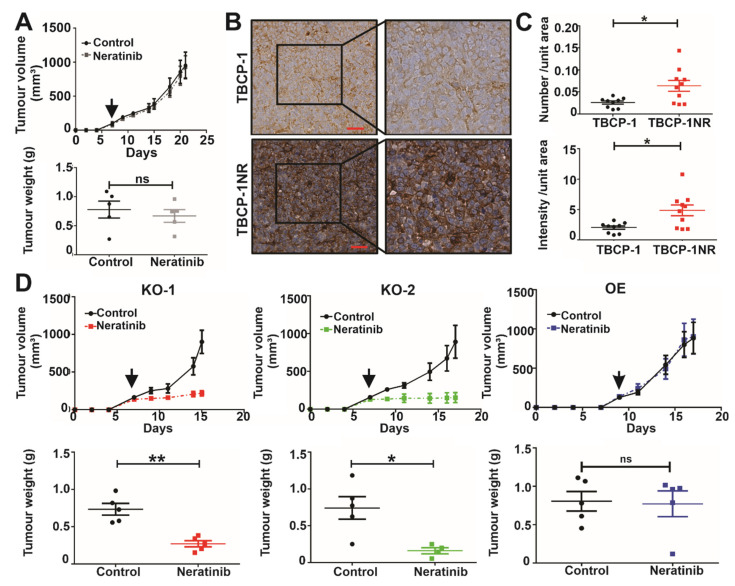
Integrin β3 regulates neratinib sensitivity in vivo. (**A**) Mice bearing orthotopic TBCP-1NR tumours were treated with vehicle control (*n* = 5) or neratinib (60 mg/kg, *n* = 5) daily by oral gavage starting when tumours reached 100 mm^3^ (indicated by an arrow). Data show mean tumour volume of control or neratinib (60 mg/kg) treated mice ± SEM (top panel) and mean tumour weights ± SEM at endpoint (bottom panel). Statistical significance was determined using an unpaired *t*-test, *p* < 0.05 was considered significant. ns = not significant. (**B**) Expression of integrin β3 was analysed in TBCP-1 (*n* = 9) and TBCP-1NR (*n* = 10) primary tumours using standard IHC. The figure shows representative images of stained TBCP-1 or TBCP-1NR primary tumours. Red (Scale bar) = 50 μm. (**C**) The expression of integrin β3 was quantitated on Aperio scanned slides using the positive pixel macro in Imagescope software. Data show mean number (top panel) and intensity (bottom panel) of strong positive pixels per unit area ± SEM. Statistical significance was determined using Mann–Whitney *t*-test, *p* < 0.05 considered significant, * *p* < 0.05. (**D**) Mice bearing orthotopic integrin β3 KO TBCP-1NR tumours (clone KO-1, clone KO-2 or clone KO-1+ integrin β3 OE) were treated with vehicle control or neratinib (60 mg/kg) daily by oral gavage starting when tumours reached 100 mm^3^ (indicated by an arrow). Data show reduced tumour growth rate after neratinib treatment in KO-1 or KO-2 tumours but not in KO-1 tumours with forced integrin β3 OE (top panel). These trends were also represented in the primary tumour weights measured at endpoint. Data (bottom panel) show mean weight ± SEM. Statistical significance was determined using Mann–Whitney *t*-test, *p* < 0.05 was considered significant, * *p* < 0.05, ** *p* < 0.01, ns = not significant.

We further characterised the growth and neratinib response of TBCP-1NR tumours in vivo. TBCP-1NR cells were inoculated in the mammary fat pad and the mice were treated daily with vehicle control or neratinib (60 mg/kg) by oral gavage for 17 days, starting when tumours were palpable. This dose was shown previously to inhibit tumour growth and metastasis in sensitive TBCP-1 tumours [23]. In agreement with our observations in vitro (Table 1), TBCP-1NR tumours maintained their resistance to neratinib in vivo (Figure 6A). Moreover, quantitative IHC analysis of integrin β3 expression in TBCP-1NR tumours revealed a clear increase in expression compared to TBCP-1 tumours (Figure 6B,C). To further confirm the functional importance of integrin β3 in mediating TKI resistance in vivo, TBCP-1NR β3 KO-1 or TBCP-1NR β3 KO-2 cells were inoculated into the mammary fat pad of the mice. Treatment with vehicle control or neratinib (60 mg/kg) by oral gavage was initiated when the tumours were approximately 100 mm^3^ in size and continued until control tumours reached 1000 mm^3^ (endpoint). Remarkably, KO of integrin β3 in TBCP-1NR cells re-sensitised tumours to neratinib treatment in vivo, as indicated by reduced primary tumour growth and tumour weights at the endpoint (Figure 6D). In contrast, similar experiments assessing neratinib sensitivity in tumours from TBCP-1NR β3 KO-1 with forced OE of β3 integrin showed lack of response to neratinib and no significant difference in tumour growth or weight at endpoint between control and neratinib-treated mice (Figure 6D). Taken together, these observations provide “proof of principle” that integrin β3 could be targeted to reverse TKI resistance in vivo. 

## 4. Discussion

TKIs provide an attractive treatment option for advanced HER2-positive breast cancer but resistance to monotherapies is common and negatively affects patient outcomes. The aim of this study was to develop and characterise new models of neratinib resistance and to identify key molecular mechanisms involved in the promotion of, and resistance to TKI-induced ferroptosis in HER2-positive breast cancer. Our findings show that the mouse and human models of neratinib resistance also acquire cross-resistance to other HER2-targeting TKIs and to ferroptosis inducers. These observations are consistent with a previous report of cross-resistance to trastuzumab and lapatinib in models of neratinib resistance [58]. However, to our knowledge, this is the first evidence that cross-resistance to the potent anti-HER2 TKI tucatinib (recently approved for HER2-positive breast cancer brain metastases) can occur. Furthermore, our observations highlight the close relationship and important functional interplay between TKI and ferroptosis resistance. Collectively, the results presented herein demonstrate the relevance of these new models of neratinib resistance and their utility to identify key alterations and vulnerabilities that could be targeted to overcome resistance to multiple HER2-targeting inhibitors. 

Notably, we found that resistance to TKI-induced ferroptosis is strongly correlated with enhanced expression of αvβ3 integrin. Accordingly, resistant cells were also associated with increased adhesion and migration to the αvβ3 integrin-binding ECM protein, VN. Furthermore, we observed a significant correlation between integrin β3 gene copy number and TKI resistance in multiple breast cancer lines by in silico analysis using the DepMap portal. The β3^high^ brain-metastatic HER2-positive MCF7-HER2Br3 cells were also highly resistant to neratinib treatment compared to β3^low^ SUM190Br and JIMT1Br3 cells. Collectively, these observations identify αvβ3 integrin protein as a potential biomarker of TKI/ferroptosis resistance. However, further studies in large cohorts of HER2-positive patients with known treatment history and clinical outcomes will be required to further validate its utility to improve patient stratification or as a predictive biomarker of treatment response. Importantly, our observations also established a critical functional link between integrin β3 expression and ferroptosis/TKI resistance. We showed that KO of this receptor in TKI-resistant mouse TBCP-1NR cells in vitro and tumours in vivo or in human MCF7-HER2Br3 cells in vitro is sufficient to reverse resistance to multiple TKIs. Conversely, forced overexpression of integrin β3 in the integrin β3 KO mouse cells and tumours or in β3^low^ human SKBR3 and BT474 cells re-instated neratinib resistance. These results support and extend previously published reports showing an association between integrin αvβ3 signalling and TKI resistance in breast cancer, lung cancer, and melanoma models [59,60,61]. Recently, Endo and colleagues [62] also demonstrated the important role of integrin αv in promoting resistance to the HER2-targeting antibody–drug conjugate, TDM1. However, while this study established a functional link between EGFR and αv integrin in regulating cell invasive properties in vitro in TDM1-resistant cells, whether genetic or pharmacological targeting of αvβ3 receptor could reverse TDM1 resistance remains to be tested. Our findings that KO or pharmacological inhibition of β3 integrin with Disba-01 is sufficient to restore sensitivity to multiple TKIs/ferroptosis inducers support this possibility and warrant further investigation of its efficacy in combination with antibody–drug conjugates such as TDM1. It is noteworthy that reversal of neratinib resistance was observed both in vitro and in vivo and that β3 KO alone did not alter significantly cell proliferation in vitro or tumour growth kinetics in vivo. These observations indicate that the reversal of neratinib resistance by β3 KO in vivo is unlikely to be mediated through β3-dependent changes in tumour angiogenesis. Integrin αvβ3 has been implicated also in promoting cancer stemness and resistance to chemotherapy in breast and ovarian cancer models [63,64,65]. Conceivably, “αvβ3 integrin addiction” may represent an Achilles’ heel that could be targeted pharmacologically to prevent or reverse resistance to multiple cancer therapies. 

The αvβ3 integrin inhibitor, Cilengitide, used herein was not evaluated in vivo due to its short half-life and poor pharmacodynamic properties [66,67]. It is noteworthy that most of the RGD-based anti-αvβ3 integrin antagonists under clinical evaluation such as Cilengitide are competitive inhibitors that interfere with ECM ligand binding. Paradoxically, at sub-optimal doses, these inhibitors act as partial agonists that can promote cell adhesion and angiogenesis, which can limit their anti-tumour response in vivo [68]. Given that integrin β3-mediated drug resistance has been shown previously to be ligand-independent [59], future studies may benefit from the evaluation of pure αvβ3 antagonists that are ligand-independent and do not induce receptor activation at lower concentrations [69,70]. Disba-01, in addition to its direct anti-tumour properties, mediates potent anti-angiogenic activity via the blockade of αvβ3-VEGFR2 crosstalk [71]. In addition, the ability of Disba-01 to downregulate αvβ3 expression (Figure 4E) would be expected to block downstream signalling and contribute to its greater potency, compared to Cilengitide. In contrast to Cilengitide, Disba-01 has been shown to induce autophagy in breast tumour cells [72], a process closely associated with the degradation of ferritin (ferritinophagy) and ferroptosis [73,74]. Thus, it is tempting to speculate that Disba-01 could synergistically enhance neratinib-induced ferroptosis in part by increasing intracellular labile iron pools and/or interfering with downstream signalling intermediates, leading to suppression of System Xc-, as observed in cells lacking β3 integrin (Figure 5H). These possibilities are currently being investigated. Overall, our findings using integrin β3 KO/OE models in vitro and in vivo provide important proof that this receptor is a relevant therapeutic target. Given the well-documented role of integrin β3 in modulating immune response [75,76], future studies should also investigate whether changes in the immune microenvironment of tumours lacking integrin β3 expression may also influence in part response to TKI therapy.

Interestingly, we found that TKI-resistant cells acquire a mesenchymal-like morphology and develop a spindle-like shape with prominent actin stress fibres. Importantly, actin cytoskeleton re-organisation was directly linked to integrin β3 expression since genetic KO of integrin β3 abolished these changes, while forced OE re-instated the elongated mesenchymal-like phenotype. These observations are also in line with previous reports showing that enhanced αvβ3 integrin expression is associated with the acquisition of an elongated morphology, loss of cell–cell contact, epithelial to mesenchymal transition (EMT), and increased migration and invasion capacity in drug-resistant cells [46,77,78,79,80]. However, whether the observed morphological changes in our models are accompanied by changes in the key EMT effectors involved in drug resistance [80,81,82,83] remains to be determined. It is noteworthy that TBCP-1NR, in addition to being more adhesive and migratory, unexpectedly showed increased proliferation suggesting that the cells may be phenotypically plastic and/or adopt a hybrid epithelial–mesenchymal state that is more favourable for metastasis and drug resistance, and characteristic of cancer stem cells [84]. 

Integrins are known to interact with RTKs and influence downstream signalling [27,85]. Importantly, αvβ3 integrin-dependent AKT activation has been shown to directly influence therapy resistance [38,86]. In TKI-resistant models, we found that a high expression of integrin β3 was associated with the suppression of HER2-EGFR-ERK signalling and persistent AKT activation. Importantly, KO of integrin β3 or its pharmacological inhibition reversed this effect and re-sensitised the cells to neratinib-induced AKT inhibition while forced β3 OE re-instated persistent AKT activation. These results highlight the important contribution of sustained AKT activation in mediating αvβ3 integrin-dependent resistance to neratinib-induced ferroptosis and to other TKIs. Future investigation will be required to determine whether this activation is mediated through a focal adhesion kinase-SRC dependent axis or other signalling effects that have been implicated in integrin β3-AKT signalling [61,87]. However, our findings show that direct inhibition of AKT using AKT inhibitor VIII, while inhibiting tumour cell proliferation and viability at high concentration (2 µM), is not sufficient at a lower concentration to synergistically reverse neratinib resistance in TBCP-1NR cells in vitro. It is important to note that the concentration of AKT inhibitor VIII required to observe synergy in combination with neratinib in TBCP-1NR cells was almost two-fold higher than its IC50 (1.37 µM). These observations are also consistent with the limited clinical activity of AKT inhibitor monotherapy in patients with advanced breast cancers [88,89,90]. While the combination of AKT or PI3K inhibitors (upstream of AKT signalling) with HER2-targeting therapies provides some clinical benefit, it is associated with high systemic toxicity [91,92,93]. Thus, induction of ferroptotic cell death by AKT/HER2 inhibitor combinations in resistant HER2-positive breast tumours may be difficult to achieve without undesired adverse effects in patients. High expression and accessibility of cell surface αvβ3 integrin receptors may provide a more promising alternative. 

Recent studies have shown that manipulation of ferroportin-1 (iron export protein) expression can alter cell sensitivity to ferroptosis [22,24]. In line with this, we found that neratinib resistance (and cross-resistance to other TKIs) is associated with increased expression of ferroportin-1 in mouse and human models of HER2-positive breast cancer, which may represent a metabolic adaptation to protect resistant cells from iron overload and excessive oxidative stress. We were also interested in clarifying whether these changes were regulated by, or independent of integrin β3 upregulation. Our observations in the TBCP-1 model indicate that the expression of ferroportin-1 decreased following integrin β3 KO, suggesting a functional link between integrin β3 signalling and iron homeostasis. However, we noted that integrin β3 OE did not completely restore ferroportin-1 expression, indicating that high β3 expression alone is not sufficient to increase ferroportin-1 expression and/or that enhanced ferroportin-1 activity and high β3 expression in resistant cells may both contribute to resistance independently. Importantly, ferroportin-1 KO alone did not restore neratinib sensitivity, likely due to compensatory changes in the expression of other iron metabolic effectors such as ferritin and TFR1 (Supplementary Figure S7E), indicating that this approach is unlikely to be sufficient to improve therapy response. Thus, integrin β3 inhibition is likely to be a more effective strategy to target TKI-resistant disease. 

Ferroptosis is a tightly regulated process driven primarily by the cystine-glutamate antiporter System Xc-, which imports cystine for the synthesis of GSH, itself required for the activity of GPX4 [94]. Previously, the pan-TKI sorafenib (which does not target HER2) was shown to act also by inhibition of System Xc- [95]. Our findings show for the first time that neratinib non-competitively inhibits the activity of System Xc- and that neratinib-induced ferroptosis can be prevented by the addition of NAC. In contrast, inhibition of cystine-uptake was not observed in cells treated by lapatinib, a less potent TKI that does not induce ferroptosis alone [24]. On that basis, it is tempting to speculate that System Xc- inhibitors may be useful to enhance the activity of non-ferroptotic TKIs and achieve similar potency to that of neratinib. We found also that the expression of System Xc- subunit SLC3A2 was moderately but consistently enhanced in TKI-resistant cells. This was accompanied by an increased rate of cystine uptake in resistant cells. Our work provides a mechanistic model through which neratinib-induced inhibition of System Xc- could explain its superior activity compared to other HER2-targeting TKIs. Work is underway to determine if neratinib inhibits System Xc- directly or via signalling intermediates. Of relevance, the transmembrane and cytoplasmic domain of SLC3A2 interacts with integrins, including αvβ3 integrin and influences their signalling [56,57,96]. Hence, we propose that changes in System Xc- expression and/or activity in TKI-resistant cells may be driven in part by β3 integrin upregulation. In support, KO of integrin β3 reversed the increase in SLC3A2 expression and decreased the uptake of cystine, while forced integrin β3 OE reversed these changes. Similarly, higher expression of β3 integrin in TBCP-1NR compared to parental TBCP-1 cells was associated with increased cystine uptake (Figure 5H) and elevated GSH levels (Supplementary Figure S6I). These findings are also consistent with a previous study in human laryngeal carcinoma cells studies indicating that αvβ3 integrin confers resistance to chemotherapeutic drugs through GSH-dependent elimination of ROS [97]. Taken together, these data indicate that crosstalk between integrin β3 and the antioxidant machinery contributes to the acquisition of resistance to TKI-induced ferroptosis. 

We do not currently know the precise mechanism by which neratinib-resistant cells or tumours upregulate integrin β3. We hypothesise that the nuclear factor erythroid 2–related factor 2 (Nrf2) transcription factor may play a central role in this process. Nrf2 is a critical regulator of cellular defence against oxidative stress through the coordinated expression of several antioxidant genes including System Xc- as well as integrin β3 [98,99]. Moreover, recent studies have established that Nrf2 also regulates iron metabolism and plays an important role in protection from ferroptosis [100,101]. Hence, we predict that the functional cooperation between Nrf2 and its target genes will be involved in coordinating the expression and function of integrin β3, System Xc- and ferroportin-1, thereby dictating cellular response to ferroptosis-inducing therapies. This possibility is currently under investigation. Unexpectedly, the expression of GPX4 was not significantly decreased in two out of three neratinib-resistant models. These observations may reflect a reduced requirement for GPX4-dependent protection from ferroptosis in some resistant cells. This could be attributed to the enhanced ability to store and/or export iron due to increased expression of ferritin and/or ferroportin-1 and increased ability to import cystine for GSH synthesis upon increased expression/activity of System Xc-. Alternatively, resistant cells may rely on other GPX4-independent pathways mediated by proteins such as Ferroptosis Suppressor Protein-1 (FSP-1) [102,103]. These possibilities will be explored in future studies. 

## 5. Conclusions

We propose a model in which integrin β3-mediated resistance to TKI-induced ferroptosis is driven by persistent AKT signalling as well as crosstalk with iron and antioxidant metabolic pathways. These changes likely tip the balance towards a low oxidative stress state and protection from ROS-induced cellular damage. The precise signalling pathways regulating the functional interplay between β3 integrin, iron, and antioxidant metabolism have yet to be determined and will be explored in future studies. Importantly, our findings with pharmacological inhibitors in vitro and with integrin β3 KO/OE tumour models in vitro and in vivo demonstrate that αvβ3 integrin inhibition could be an effective strategy to prevent or reverse resistance to TKI-induced ferroptosis. 

## Figures and Tables

**Table 1:** Neratinib resistance is associated with cross-resistance to other HER2-targeting TKIs and ferroptosis inducers.

	TBCP-1			SKBR3			BT474		
DRUG *	PAR	NR	FOLD DIFFERENCE	PAR	NR	FOLD DIFFERENCE	PAR	NR	FOLD DIFFERENCE
Neratinib	0.21 ± 0.05	2.92 ± 1.01	13.9	0.009 ± 0.009	0.1 ± 0.07	11.1	0.003 ± 0.001	0.062 ± 0.065	20.6
Lapatinib	0.58 ± 0.08	>16	>27.6	0.15 ± 0.10	>4	>26.6	0.064 ± 0.001	>4	>62.5
Tucatinib	0.36 ± 0.07	>16	>44.4	0.02 ± 0.014	>4	>200	0.023 ± 0.018	>4	>173.9
Erastin	2.37 ± 0.79	6.34 ± 3.34	2.67	2.54 ± 1.73	6.38 ± 5.11	2.51	>10	>10	NA
RSL3	1.75 ± 0.04	>4	>2.29	1.39 ± 0.52	3.42 ± 1.21	2.45	1.71 ± 0.4	>10	>5.8

PAR = age-matched sensitive parental, NR = neratinib-resistant. * IC50 values indicated in μM ± SD of three independent experiments (*n* = 3), where each run had three replicate wells per inhibitor concentration.

## Data Availability

The datasets used and/or analysed during the current study are available from the corresponding author on reasonable request.

## Supplementary Materials

The following supporting information can be downloaded at: https://www.mdpi.com/article/10.3390/cancers15041216/s1. Figure S1: Changes in morphology and expression of integrin subunits in TKI resistant cells. Figure S2: Modulation of integrin β3 expression leads to coordinated changes in the expression of αv integrin and re-sensitisation to neratinib-induced ferroptosis. Figure S3: Integrin β3 functionally regulates morphology and TKI response in human HER2 positive cell lines in vitro. Figure S4: αvβ3 integrin inhibitors synergise with TKIs and ferroptosis inducers. Figure S5: Integrin β3 signals through AKT activation in TKI-resistant human HER2-positive cells. Figure S6: TKI resistance and altered integrin β3 expression are associated with changes in ferroportin-1 expression and System Xc activity in TKI-resistant cells. Figure S7: Genetic modulation of ferroportin-1 expression does not alter neratinib sensitivity. File S1: Original blots.
