# Supplementary material for: Integrin αvβ3 Is a Master Regulator of Resistance to TKI-Induced Ferroptosis in HER2-Positive Breast Cancer
Source: Cancers (Basel). 2023 Feb 14;15(4):1216. doi: 10.3390/cancers15041216 (PMC9954089; doi:10.3390/cancers15041216)
Supplement: Supplementary file 1 [file cancers-15-01216-s001.zip › cancers-2178010-Figures S1-S7.pdf]

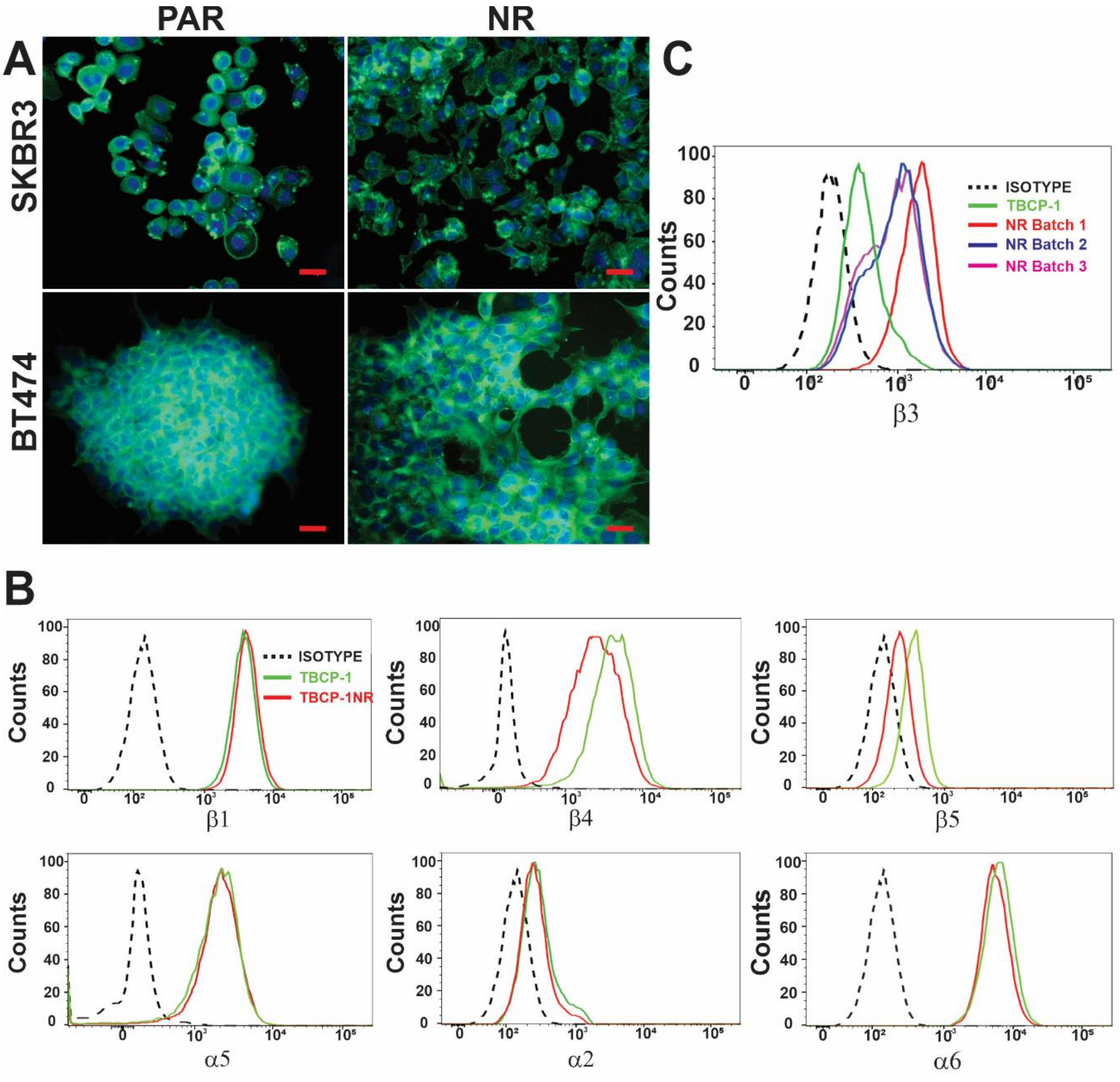

Nagpal et al 2023 Supplementary Fig. 1

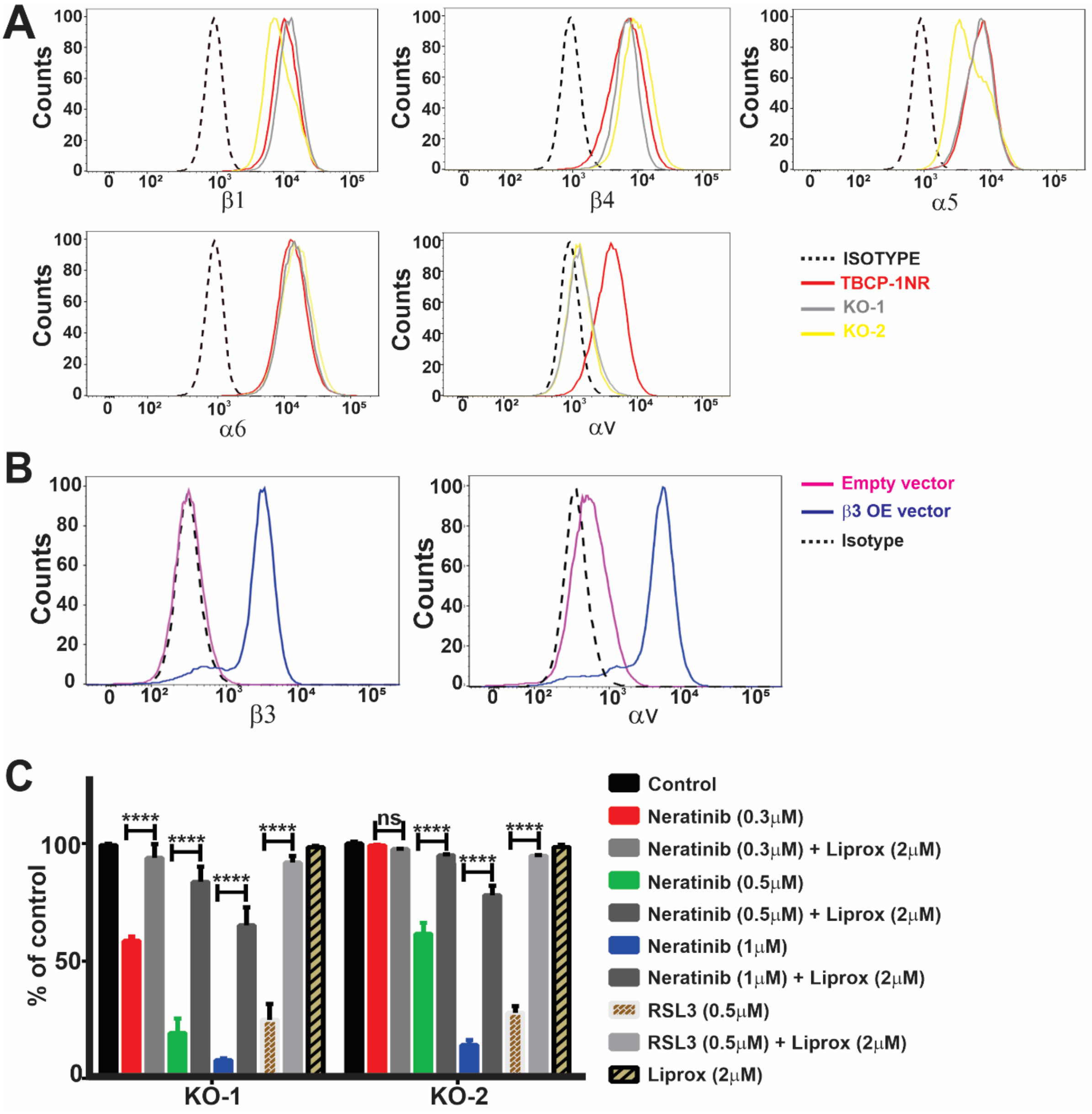

Nagpal et al 2023 Supplementary Fig. 2

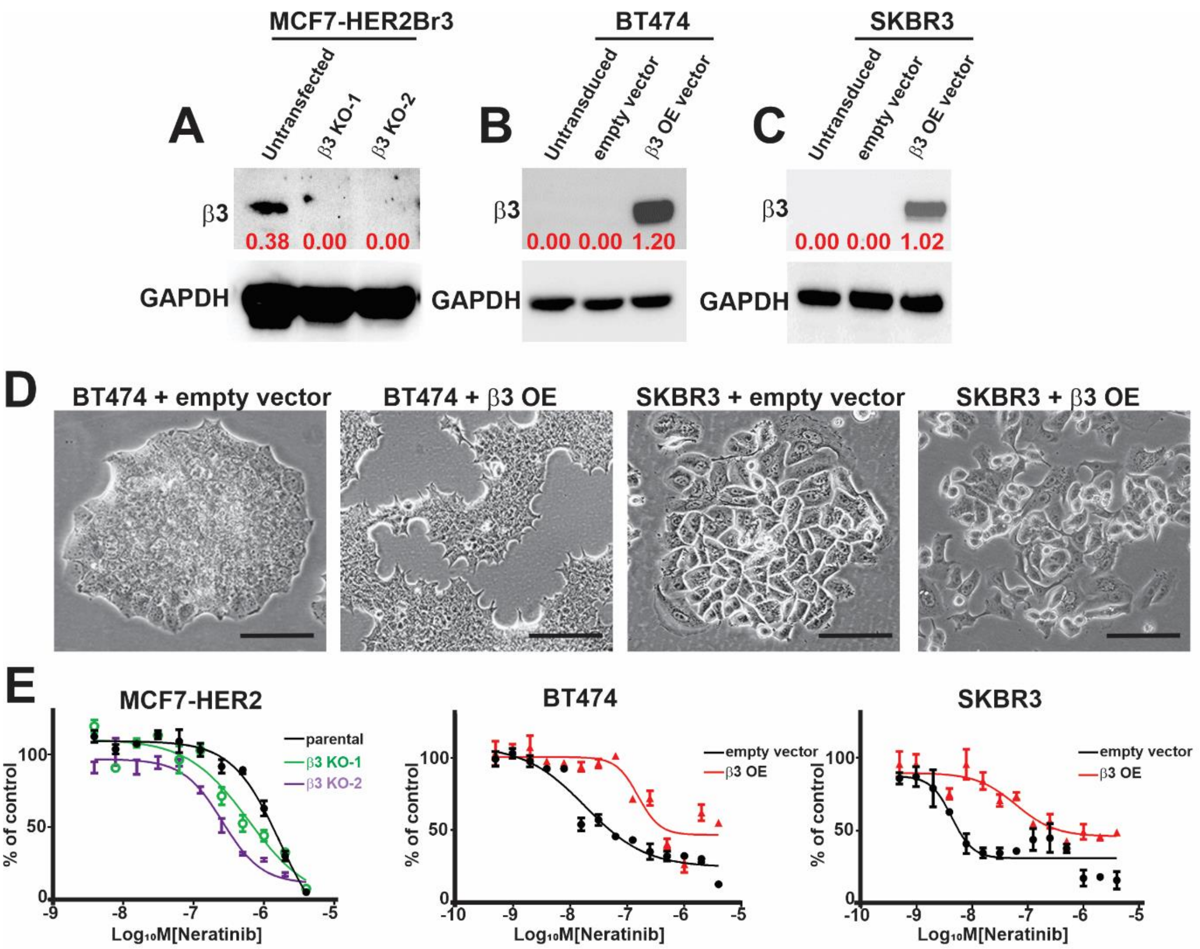

F

	MCF7-HER2Br3			BT474		SKBR3	
	parental	β3 KO-1	β3 KO-2	empty vector	β3 OE	empty vector	β3 OE
IC50* ± SD	1.41 ± 0.031	0.55 ± 0.21	0.42 ± 0.15	0.022 ± 0.007	0.138 ± 0.021	0.006 ± 0.003	0.056 ± 0.007

*IC50 values indicated in μM ± SD

Nagpal et al 2023 Supplementary Fig. 3

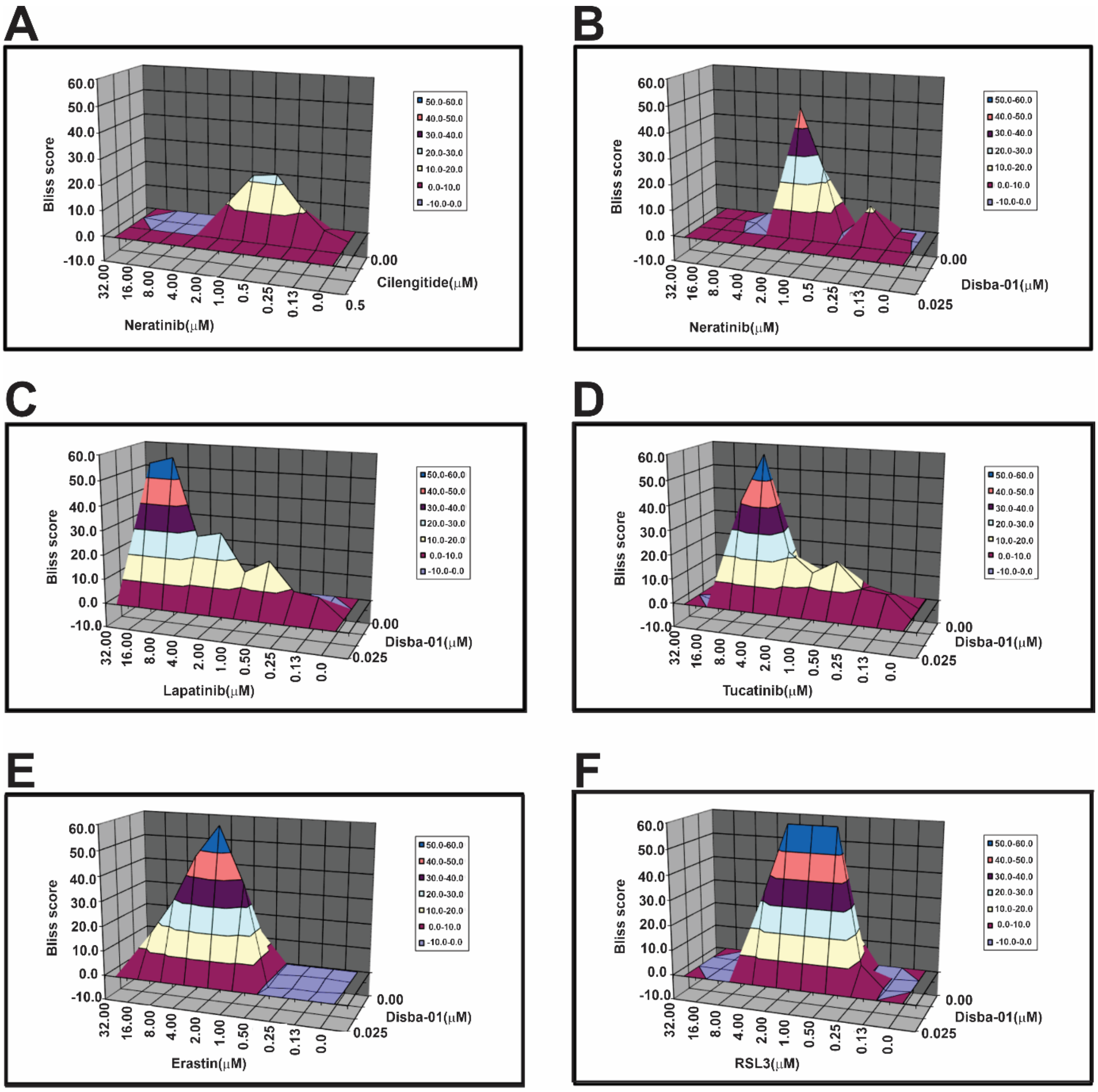

Nagpal et al 2023 Supplementary Fig. 4

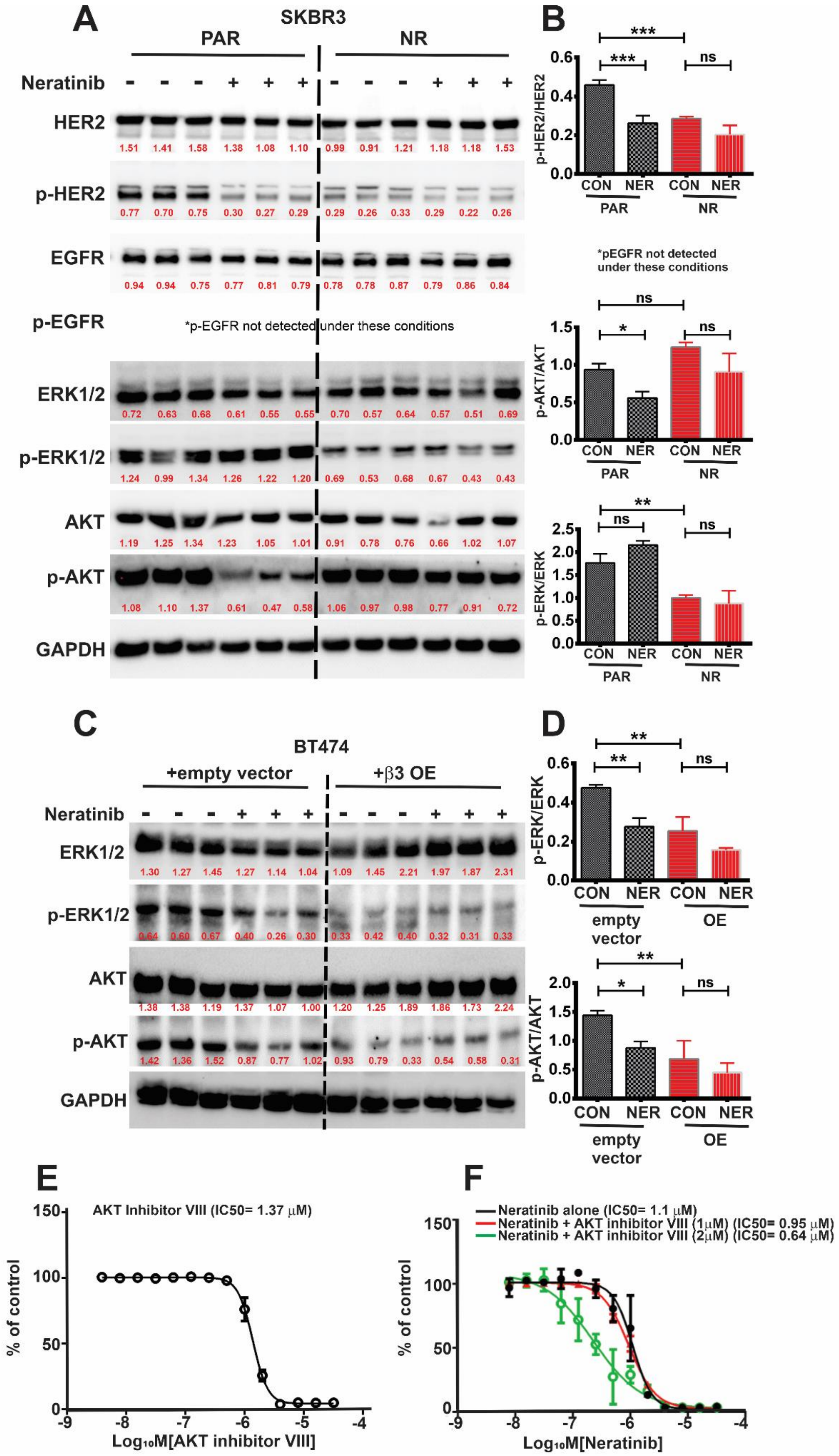

Nagpal et al 2023 Supplementary Fig. 5

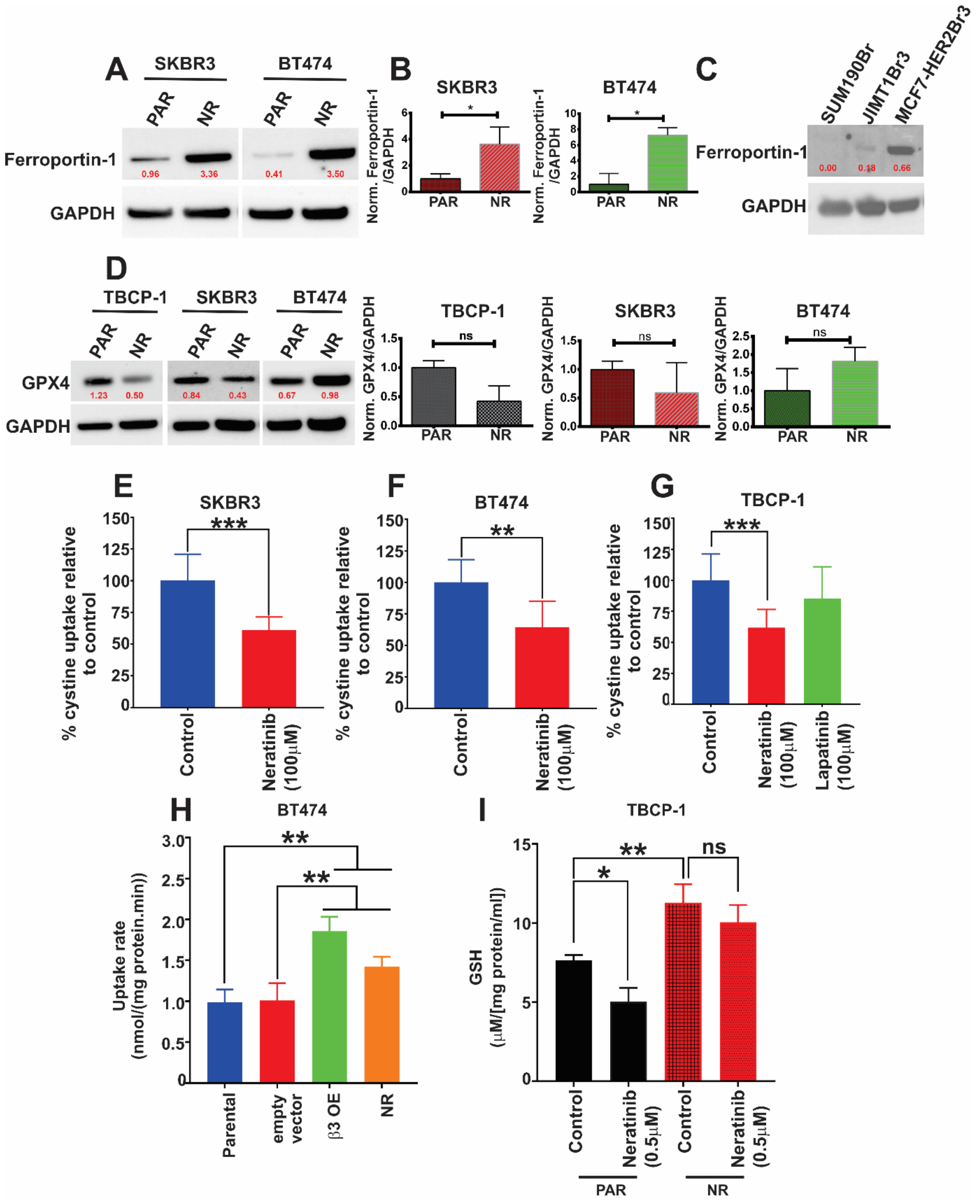

Nagpal et al 2023 Supplementary Fig. 6

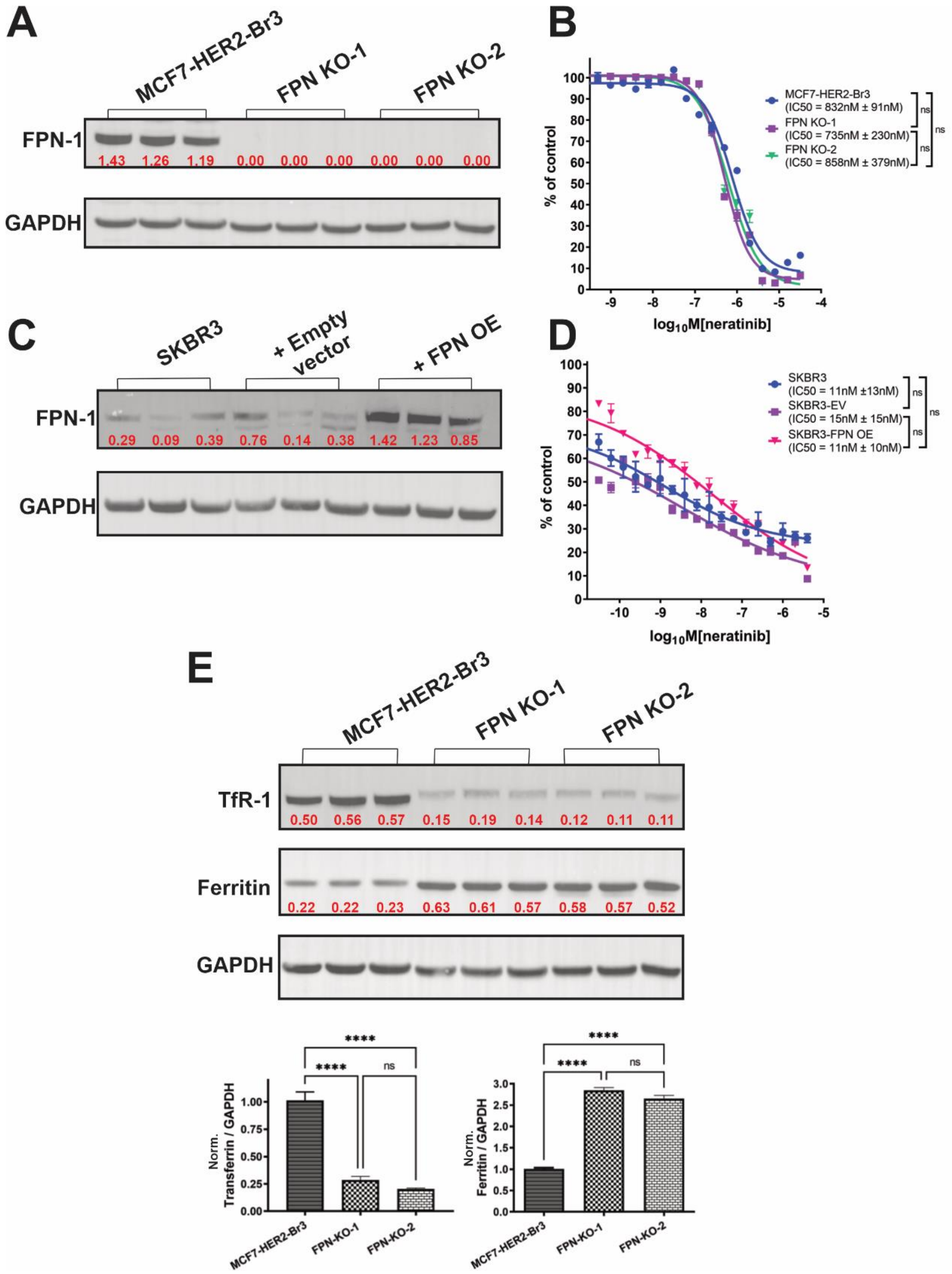

Nagpal et al 2023 Supplementary Fig. 7

Supplementary Fig. 1 Changes in morphology and expression of integrin subunits in TKI resistant cells. A The alterations in morphology and actin cytoskeleton were visualised in the human HER2 positive TKI resistant SKBR3NR and BT474NR cells (NR) and compared to age-matched sensitive SKBR3 and BT474 cells (PAR) by F-actin staining using A488-conjugated phalloidin. Red (Scale bar) = 50 μm. Blue (DAPI) = nuclei. Green (A488-phalloidin) = F-actin. **B** Cell surface expression of the indicated integrin subunits was analysed in age-matched sensitive TBCP-1 or TKI resistant TBCP-1NR cells by flow cytometry. Black dashed line = isotype control, green = TBCP-1 and red = TBCP-1NR. **C** Cell surface expression of integrin β3 was analysed in TBCP-1 cells or three independently derived batches of TBCP-1NR cells by flow cytometry. Black dashed line = isotype control, green = TBCP-1, red = TBCP-1NR batch 1, blue = TBCP-1NR batch 2 and pink = TBCP-1NR batch 3.

Supplementary Fig. 2 Modulation of integrin β3 expression leads to coordinated changes in the expression of αv integrin and re-sensitisation to neratinib-induced ferroptosis. Cell surface expression of the indicated integrin subunits was analysed cells by flow cytometry in **A** integrin β3 KO-1 and KO-2 cells **B** integrin β3 KO-1 cells transduced with empty vector or β3 OE vector. **C** Neratinib-induced cell death in integrin β3 KO clones was prevented by co-incubation with liproxstatin-1. Cells were incubated with neratinib (at the indicated doses) with or without liproxstatin-1 (2 μM) and growth relative to vehicle-treated control was determined after 72 hours using the SRB assay. The ferroptosis inducer, RSL3 (0.5 μM) was used as a positive control to confirm the anti-ferroptotic activity of liproxstatin-1. Data show mean of % growth relative to control ± SD from a representative experiment of three independent experiments (n = 3). Statistical significance was determined by two-way ANOVA Tukey's multiple comparison test, $p<0.05$ was considered significant, ****$p<0.0001$, ns= not significant.

Supplementary Fig. 3 Integrin β3 functionally regulates morphology and TKI response in human HER2 positive cell lines *in vitro*. A Genetic KO of integrin β3 was confirmed in the indicated MCF7-HER2Br3 clones by western blot analysis of whole cell lysates and GAPDH was used as the loading control. Integrin β3 OE was confirmed in td-tomato-positive FACS-sorted **B** BT474 and **C** SKBR3 cells by western blot analysis of whole cell lysates and GAPDH was used as loading control. The intensity ratio of each band relative to GAPDH is indicated in red. **D** Morphology of empty vector or integrin β3 OE vector-transduced cells was visualised by brightfield imaging. Black (Scale bar) = 50 μm **E** Sensitivity of integrin β3 KO MCF7-HER2Br3 clones or integrin β3 OE BT474 and SKBR3 cells to neratinib relative to their respective control was determined in a 3-day SRB assay. The data show representative curves and **F** mean IC50 ± SD from three independent experiments (n=3).

Supplementary Fig. 4 αvβ3 integrin inhibitors synergise with TKIs and ferroptosis inducers. The nature of the interactions between Cilengitide (0.5 μM) or Disba-01 (0.025 μM) and **A-B** Neratinib, **C** Lapatinib, **D** Tucatinib, **E** Erastin and **F** RSL3 was determined using the Bliss dose-response surface model. Synergistic interactions at each drug concentration were determined by subtracting the predicted additive cytotoxicity of the combination from the observed cytotoxicity based on the cell proliferation assays and are expressed as Bliss score on the z-axis. Bliss score > 0 indicates synergistic drug interactions, = 0 indicates additive interactions and < 0 indicates antagonistic interactions.

Supplementary Fig. 5 Integrin β3 signals through AKT activation in TKI resistant human HER2 positive cells. A The impact of neratinib treatment on EGFR-HER2 signalling was analysed in SKBR3 age-matched sensitive (PAR) and TKI-resistant (NR) cells. Changes in the levels of the indicated signalling intermediates were measured by western blot in serum-starved cells following 1 hour of vehicle control (DMSO) or neratinib (0.005 μM) treatment and 10 minutes EGF stimulation. GAPDH was used as the loading control. The intensity ratio of each band relative to GAPDH is indicated in red. **B** Data show quantitation of mean phospho/total protein ratio ± SD from three independent experiments (n=3). Statistical significance was determined by one-way ANOVA, Holm-Sidak's multiple comparison test, $p<0.05$ was considered significant, *$p<0.05$, **$p<0.005$, ***$p<0.001$, ns = not significant. **C** The impact of neratinib treatment on ERK and AKT phosphorylation was also analysed in human HER2-positive BT474 cells transduced with empty vector or integrin β3 OE vector. Changes in the levels of the indicated signalling intermediates were measured by western blot in serum-starved cells following 1 hour of vehicle control (DMSO) or neratinib (0.005 μM) treatment and 10 minutes EGF stimulation. GAPDH was used as loading control. The intensity ratio of each band relative to GAPDH is indicated in red. **D** Data show quantitation of mean phospho/total protein ratio ± SD from three independent experiments (n=3). Statistical significance was determined by one-way ANOVA, Holm-Sidak's multiple comparison test, $p<0.05$ was considered significant, *$p<0.05$, **$p<0.005$, ns=not significant. **E** The potency of AKT inhibitors VIII in TBCP-1NR cells was determined using a 3-day SRB assay. The data show the curve and IC50 from a single preliminary experiment (n=1). **F** The efficacy of combined AKT inhibitor VIII (1 μM or 2 μM) + neratinib was analysed over 3 days using an SRB assay. The data show curves and IC50 from a single experiment (n=1).

Supplementary Fig. 6 TKI resistance and altered integrin β3 expression are associated with changes in ferroportin-1 expression and System Xc activity in TKI resistant cells. A Changes in basal expression of ferroportin-1 was analysed in the age-matched sensitive (PAR) or resistant (NR) SKBR3 and BT474 cell lysates by western blot and GAPDH was used as loading control. The intensity ratio of each band relative to GAPDH is indicated in red. The data show representative blots and **B** normalised expression relative to GAPDH ± SD from three independent lysates (n=3). Statistical significance was determined using paired t-test, $p<0.05$ was considered significant, *$p<0.05$. **C** Ferroportin-1 expression was analysed in SUM190Br, JIMT1Br3 and MCF7-HER2Br3 whole cell lysates and GAPDH was used as a loading control. The intensity ratio of each band relative to GAPDH is indicated in red. **D** Changes in basal expression of GPX4 was analysed in the age-matched sensitive (PAR) or resistant (NR) mouse and human HER2-positive cell lysates by western blot and GAPDH was used as loading control. The intensity ratio of each band relative to GAPDH is indicated in red. The data show representative blots and normalised expression relative to GAPDH ± SD from three independent lysates (n=3). Statistical significance was determined using paired t-test, $p<0.05$ was considered significant, ns=not significant. Uptake of FITC-labelled cystine (200 μM) was inhibited by neratinib (100 μM) relative to control in **E** SKBR3 and **F** BT474 cells. Statistical significance was determined using the Mann-Whitney t-test, $p<0.05$ was considered significant. **$p<0.01$, ***$p<0.001$. **G** Uptake of FITC-labelled cystine (200 μM) in TBCP-1 cells was inhibited by neratinib (100 μM) but not lapatinib (100 μM) relative to the control. Statistical significance was determined using the Mann-Whitney t-test, $p<0.05$ was considered significant. *** $p<0.001$. **H** TKI resistance and integrin β3 overexpression was characterised by increased uptake of FITC-labelled cystine in human BT474 cells. Data are expressed as uptake rate (nmol/(mg protein.min)) and statistical significance was determined using the Mann-Whitney t-test, $p<0.05$ was considered significant. **$p<0.01$. **I** TKI resistance was characterised by higher GSH levels which were not reduced following neratinib treatment. Data shows mean GSH (μM/[mg protein/ml])

± SD from n=3 experiments, each performed in duplicates. Statistical analysis was performed using One-Way Anova and Tukey's multiple comparison test, $p<0.05$ was considered statistically significant. *$p<0.05$, **$p<0.01$.

Supplementary Fig. 7 Genetic modulation of ferroportin-1 expression does not alter neratinib sensitivity. **A** Knockout of ferroportin-1 expression was confirmed in the indicated MCF7-HER2Br3 clones with western blot analysis (3 independent lysates/clone) and GAPDH was used as loading control. The intensity ratio of each band relative to GAPDH is indicated in red. **B** Neratinib sensitivity of MCF7-HER2Br3 cells or ferroportin-1 KO clones was determined using a 3-day SRB assay. Data show represented curves and mean IC50 ± SD from three independent experiments. Statistical significance was determined using a Kolmogorov-smirnov t-test, ns= not significant. **C** Overexpression of ferroportin-1 was confirmed in SKBR3 cells compared to untransfected or empty vector transfected cells with western blot analysis (3 independent lysates/cell line) and GAPDH was used as loading control. The intensity ratio of each band relative to GAPDH is indicated in red. **D** Neratinib sensitivity of SKBR3 or ferroportin-1 OE cells was determined using a 3-day SRB assay. Data show represented curves and mean IC50 ± SD from three independent experiments. Statistical significance was determined using a Kolmogorov-smirnov t-test, ns= not significant. **E** Expression of iron metabolism effectors transferrin receptor (TFR1) and ferritin was analysed by western blot in whole cell lysates of MCF7-HER2Br3 cells or ferroportin-1 KO clones. The intensity ratio of each band relative to GAPDH is indicated in red. Three independent lysates per cell line were quantitated was normalised against GAPDH. Statistical significance was analysed with a one-way ANOVA and $p<0.05$ was considered significant, ****$p < 0.0001$, ns = not significant.
