# Supplementary material for: Integrin αvβ3 Is a Master Regulator of Resistance to TKI-Induced Ferroptosis in HER2-Positive Breast Cancer
Source: Cancers (Basel). 2023 Feb 14;15(4):1216. doi: 10.3390/cancers15041216 (PMC9954089; doi:10.3390/cancers15041216)

Nagpal et al 2023 Supplementary Material- Uncropped Western Blot Compilation

Figure 2B

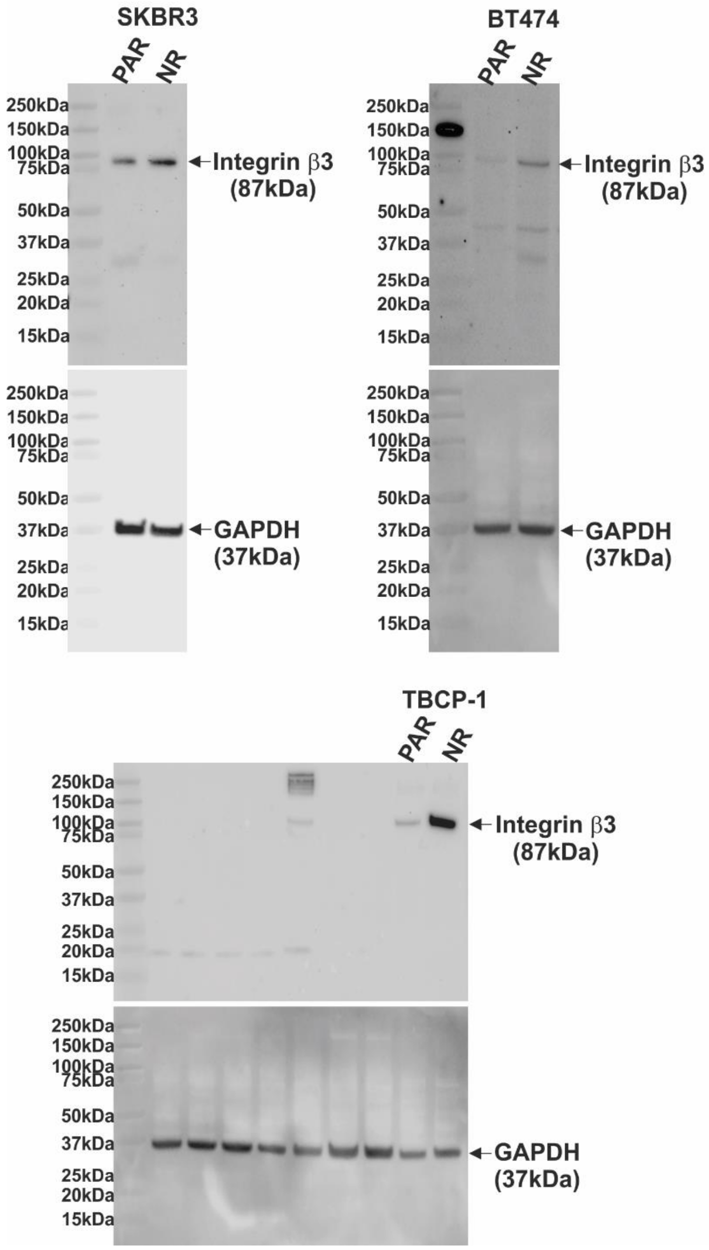

Figure 2D

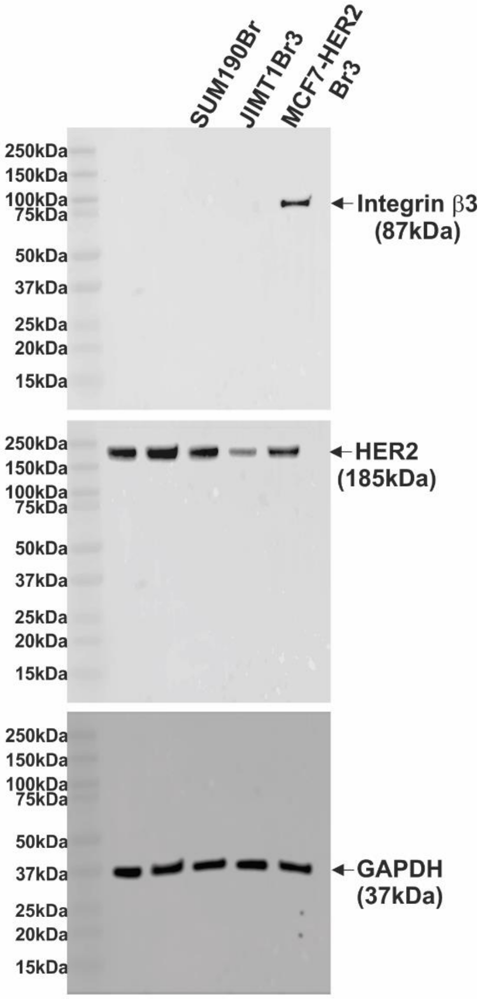

Figure 3A   Figure 3B

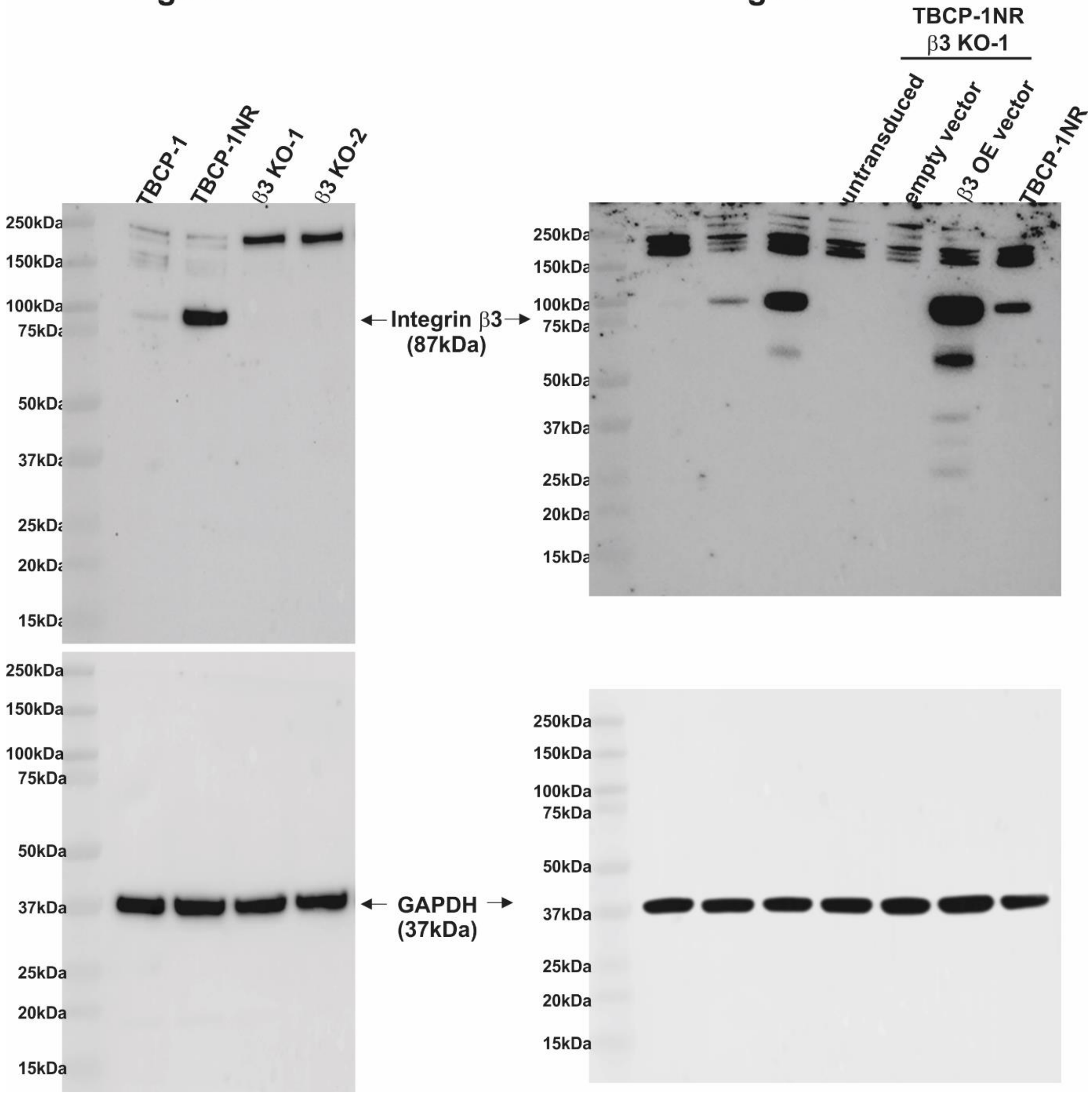

Figure 4A

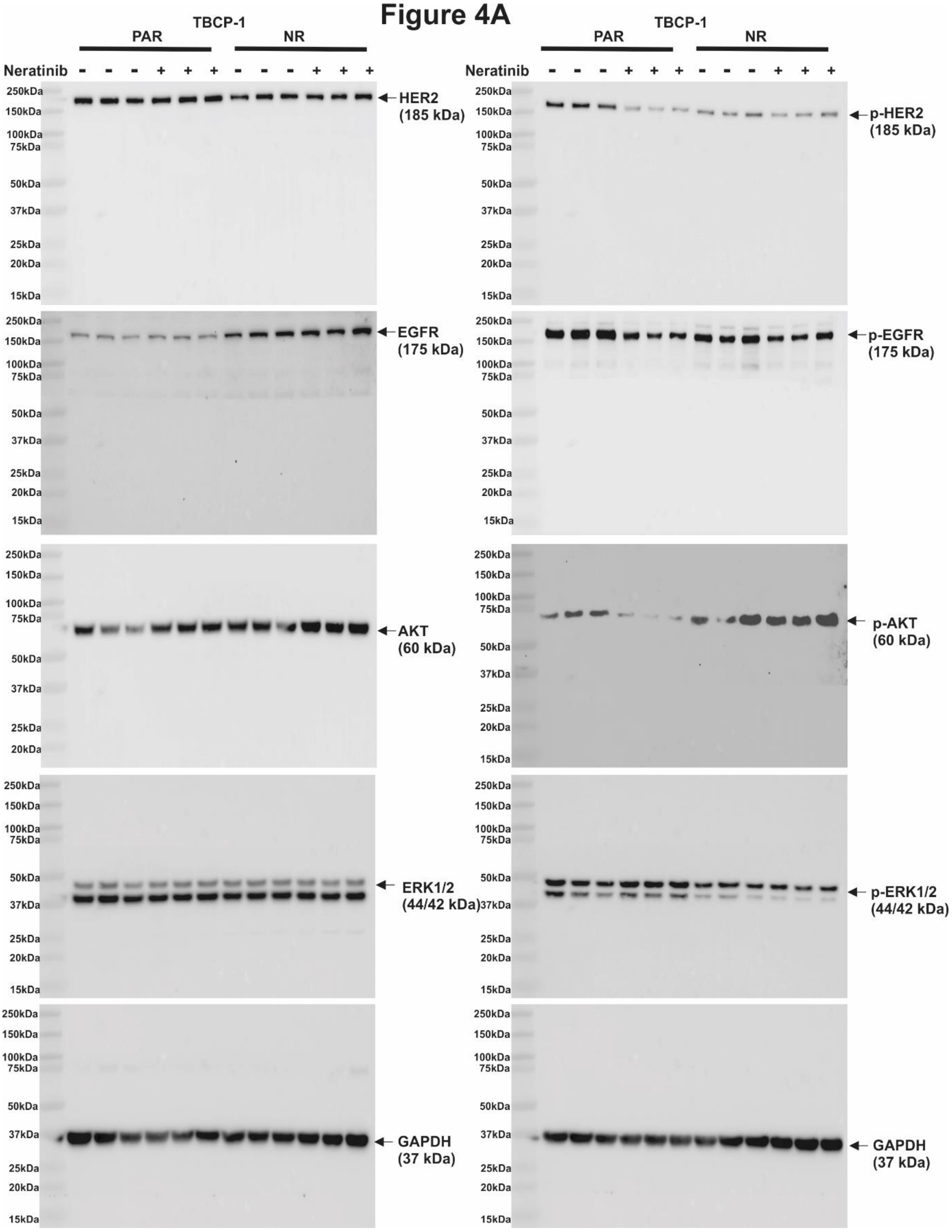

TBCP-1
PAR
NR
Neratinib
- - - + + + - - - + + +
250kDa
150kDa
100kDa
75kDa
50kDa
37kDa
25kDa
20kDa
15kDa
HER2 (185 kDa)
EGFR (175 kDa)
AKT (60 kDa)
ERK1/2 (44/42 kDa)
GAPDH (37 kDa)
p-HER2 (185 kDa)
p-EGFR (175 kDa)
p-AKT (60 kDa)
p-ERK1/2 (44/42 kDa)
GAPDH (37 kDa)

Figure 4C

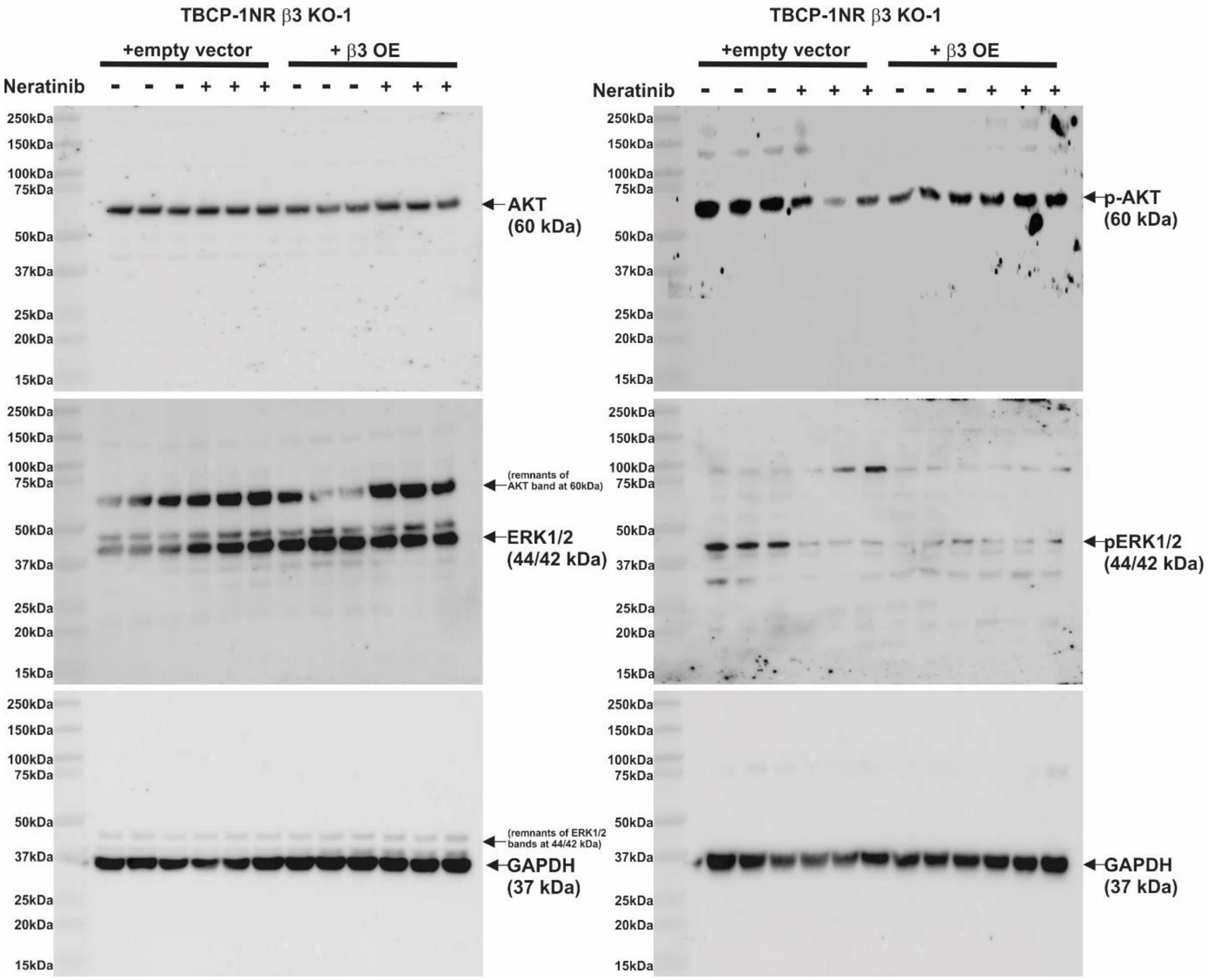
TBCP-1NR β3 KO-1
+empty vector
+ β3 OE
Neratinib - - - + + + - - - + + +
250kDa
150kDa
100kDa
75kDa
50kDa
37kDa
25kDa
20kDa
15kDa
AKT (60 kDa)
(remnants of AKT band at 60kDa)
ERK1/2 (44/42 kDa)
(remnants of ERK1/2 bands at 44/42 kDa)
GAPDH (37 kDa)
p-AKT (60 kDa)
pERK1/2 (44/42 kDa)

Figure 4E

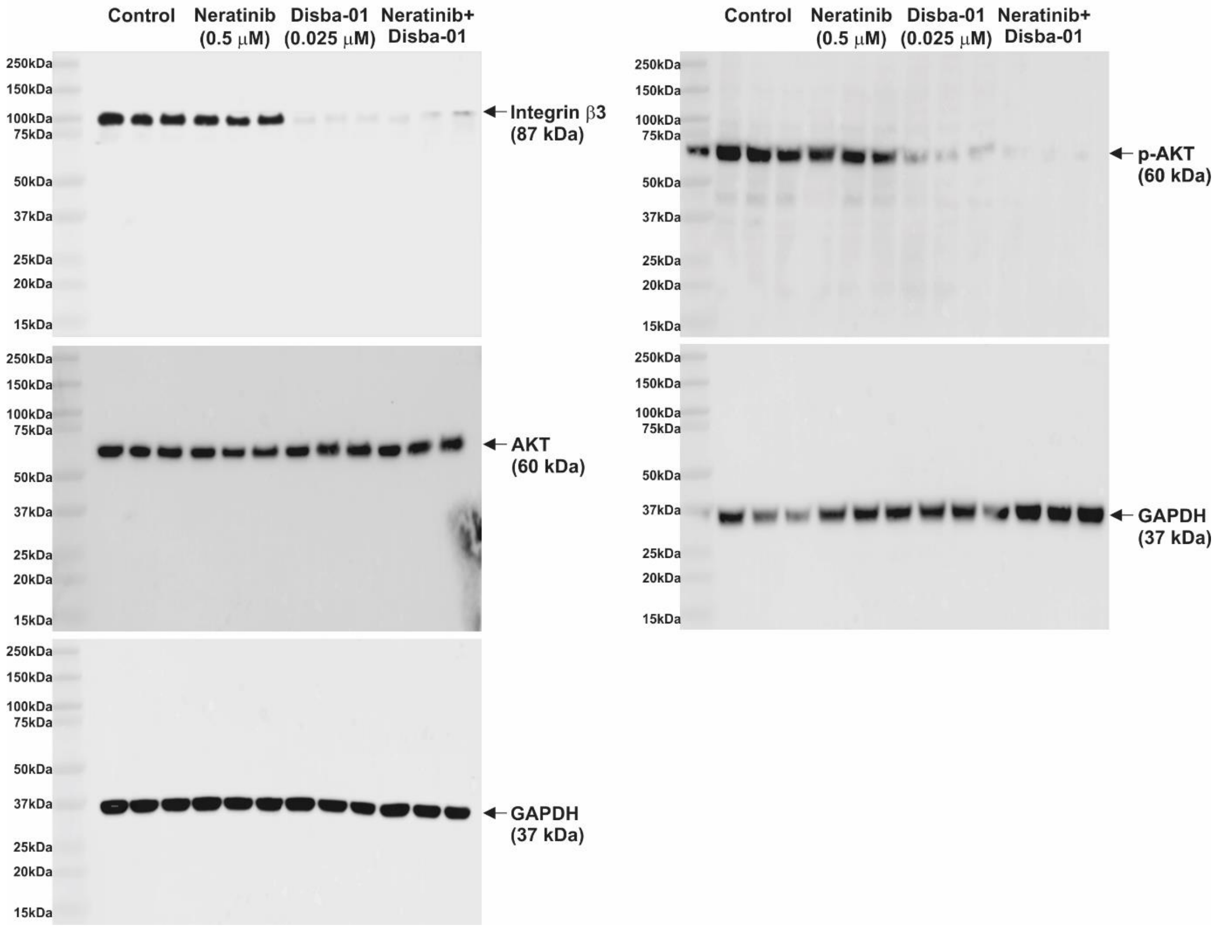
Control
Neratinib (0.5 μM)
Disba-01 (0.025 μM)
Neratinib+ Disba-01
250kDa
150kDa
100kDa
75kDa
50kDa
37kDa
25kDa
20kDa
15kDa
Integrin β3 (87 kDa)
AKT (60 kDa)
GAPDH (37 kDa)
p-AKT (60 kDa)
GAPDH (37 kDa)

Figure 5A

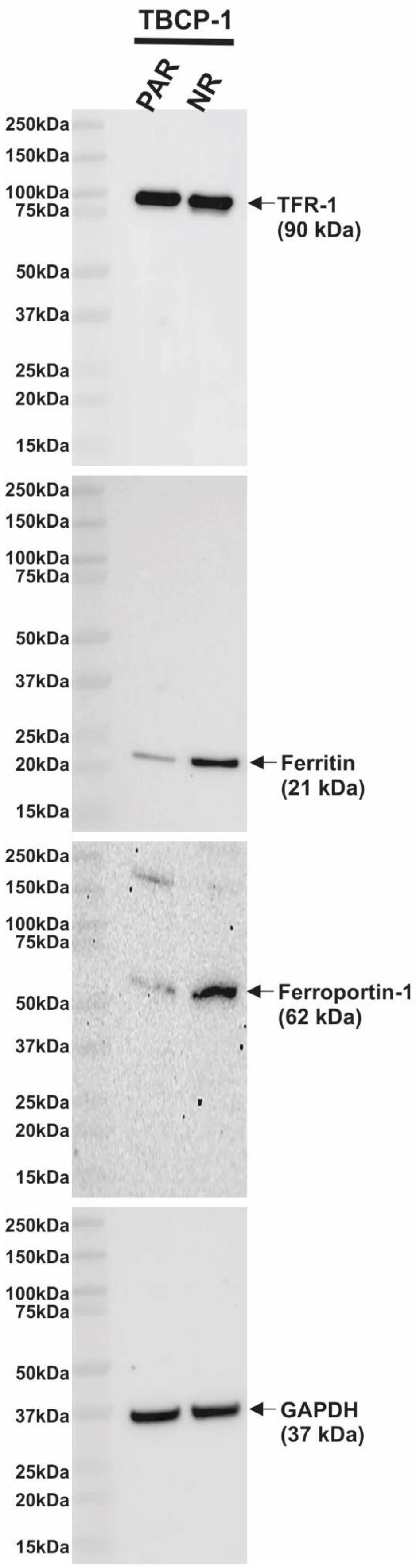

Figure 5B

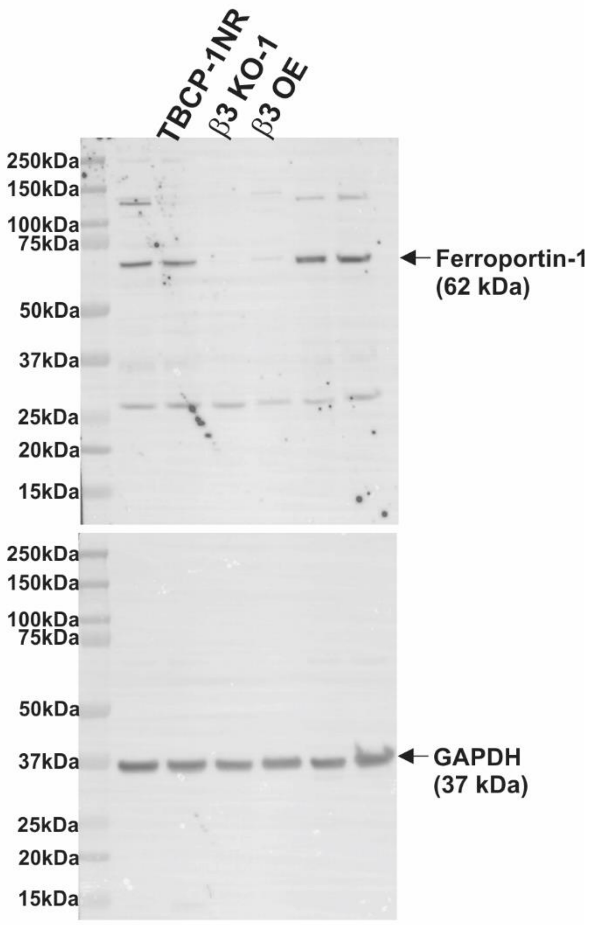

Supplementary Figure 3

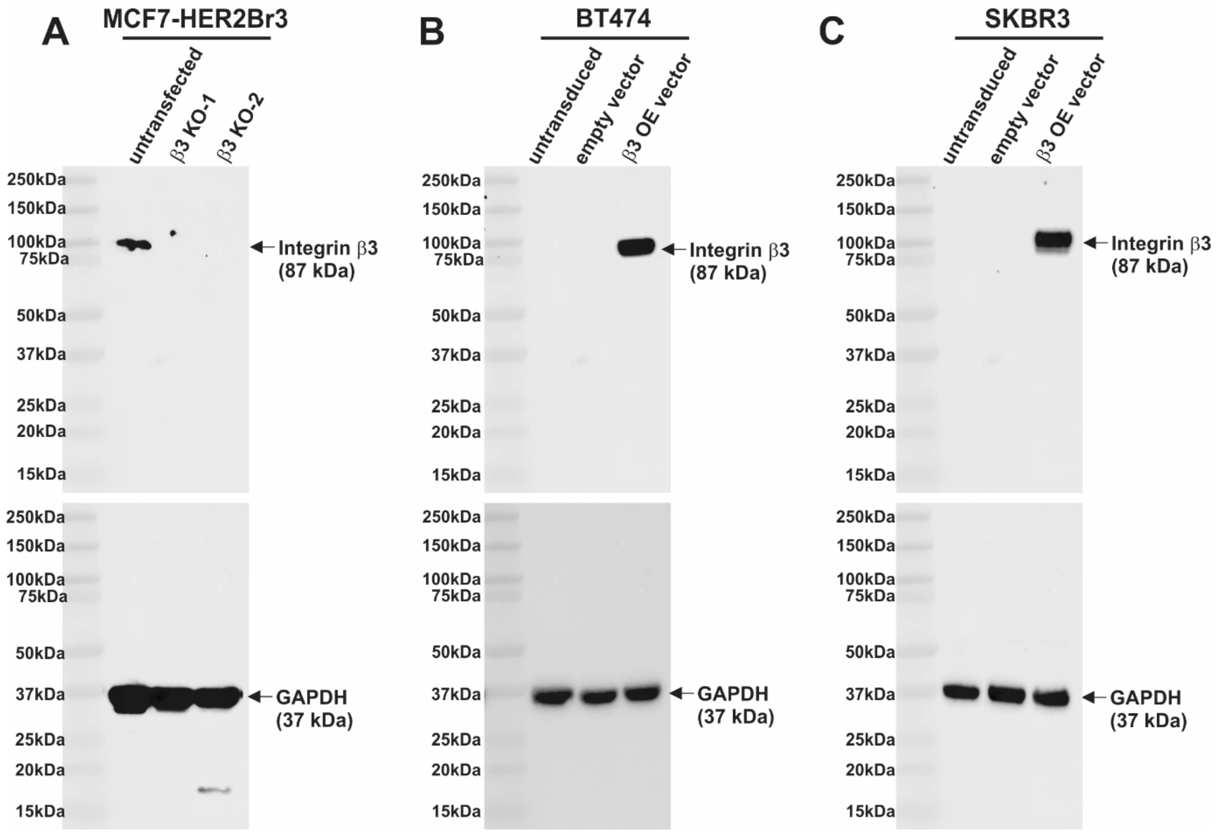

Supplementary Figure 5A

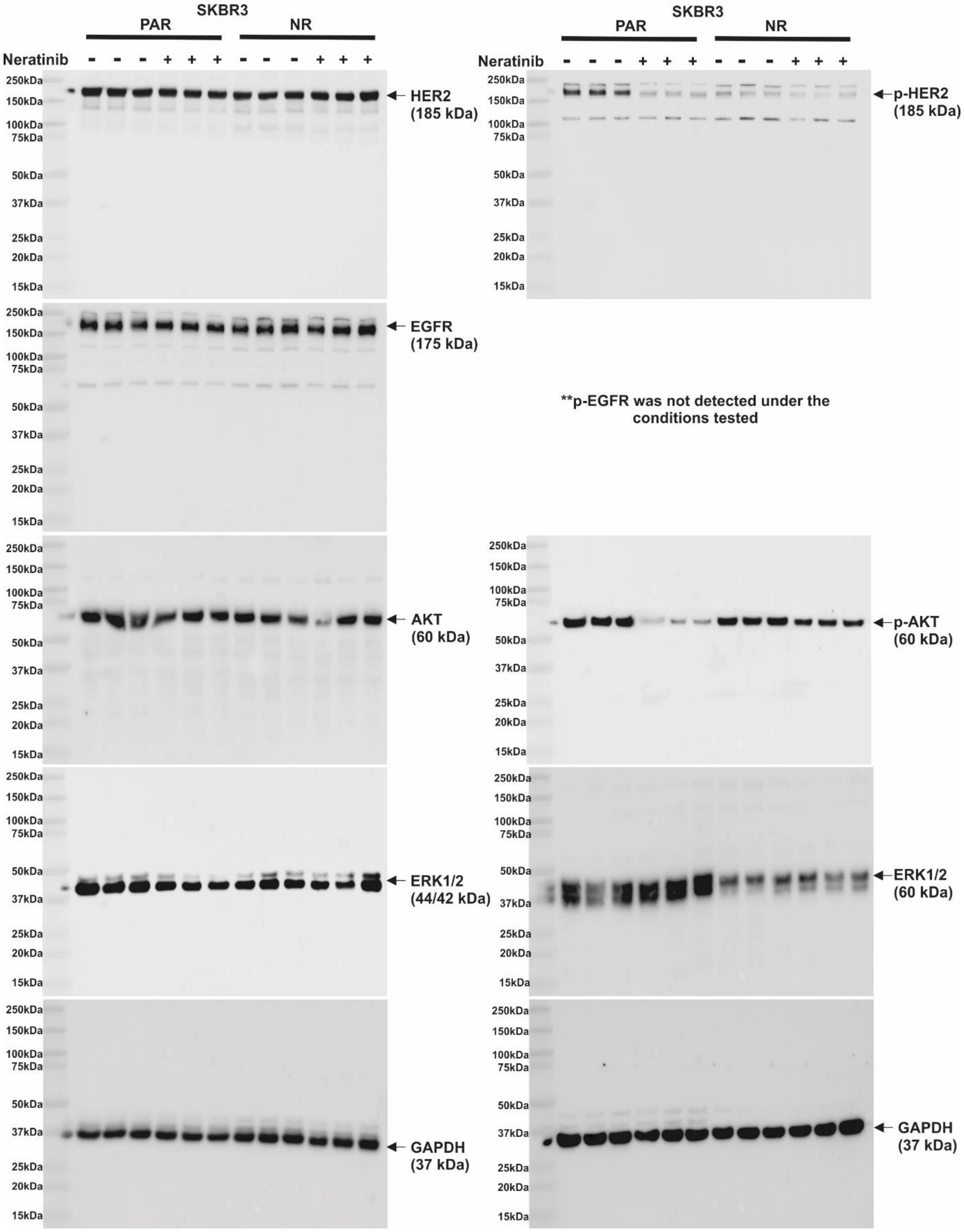

Supplementary Figure 5C

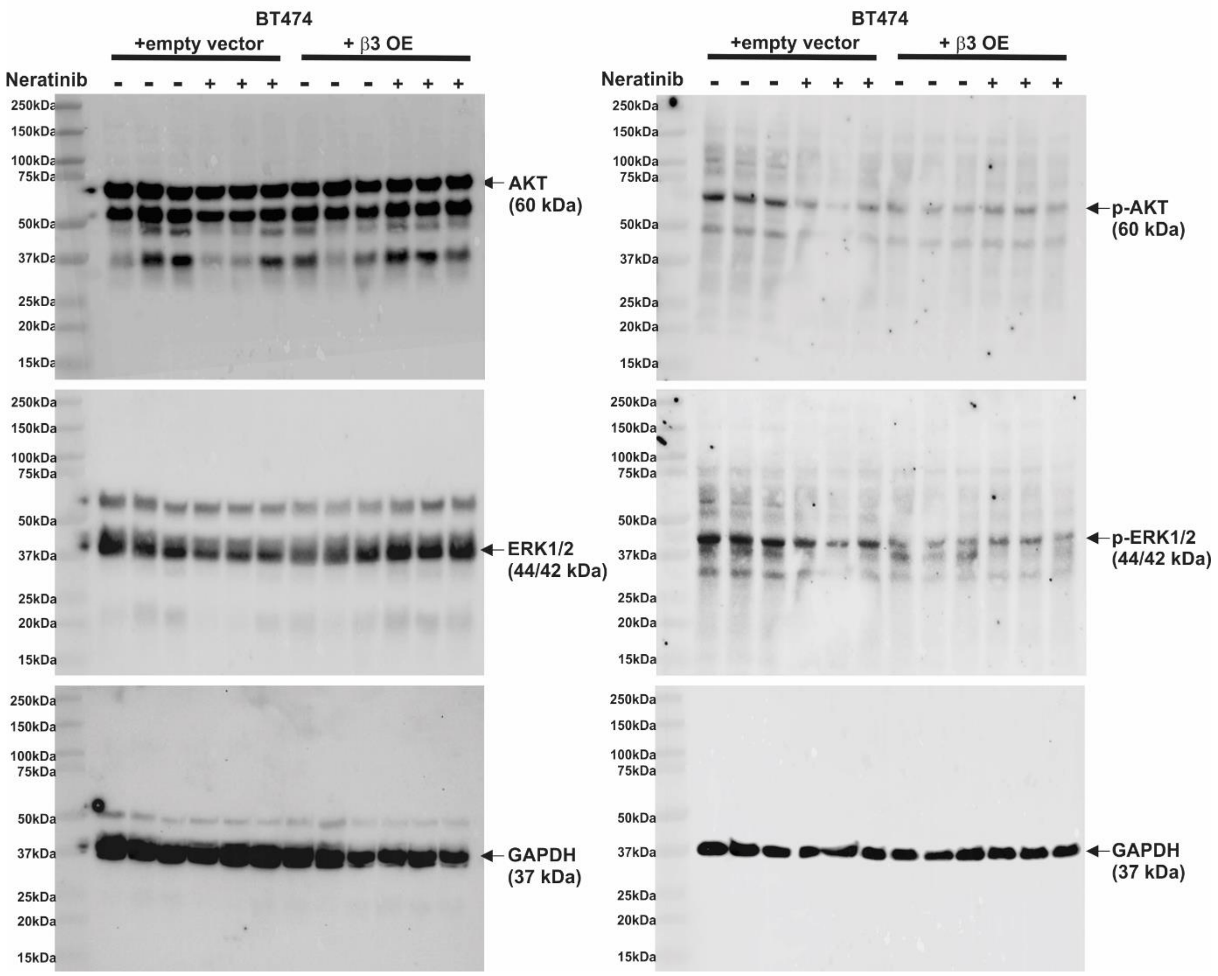

Supplementary Figure 6

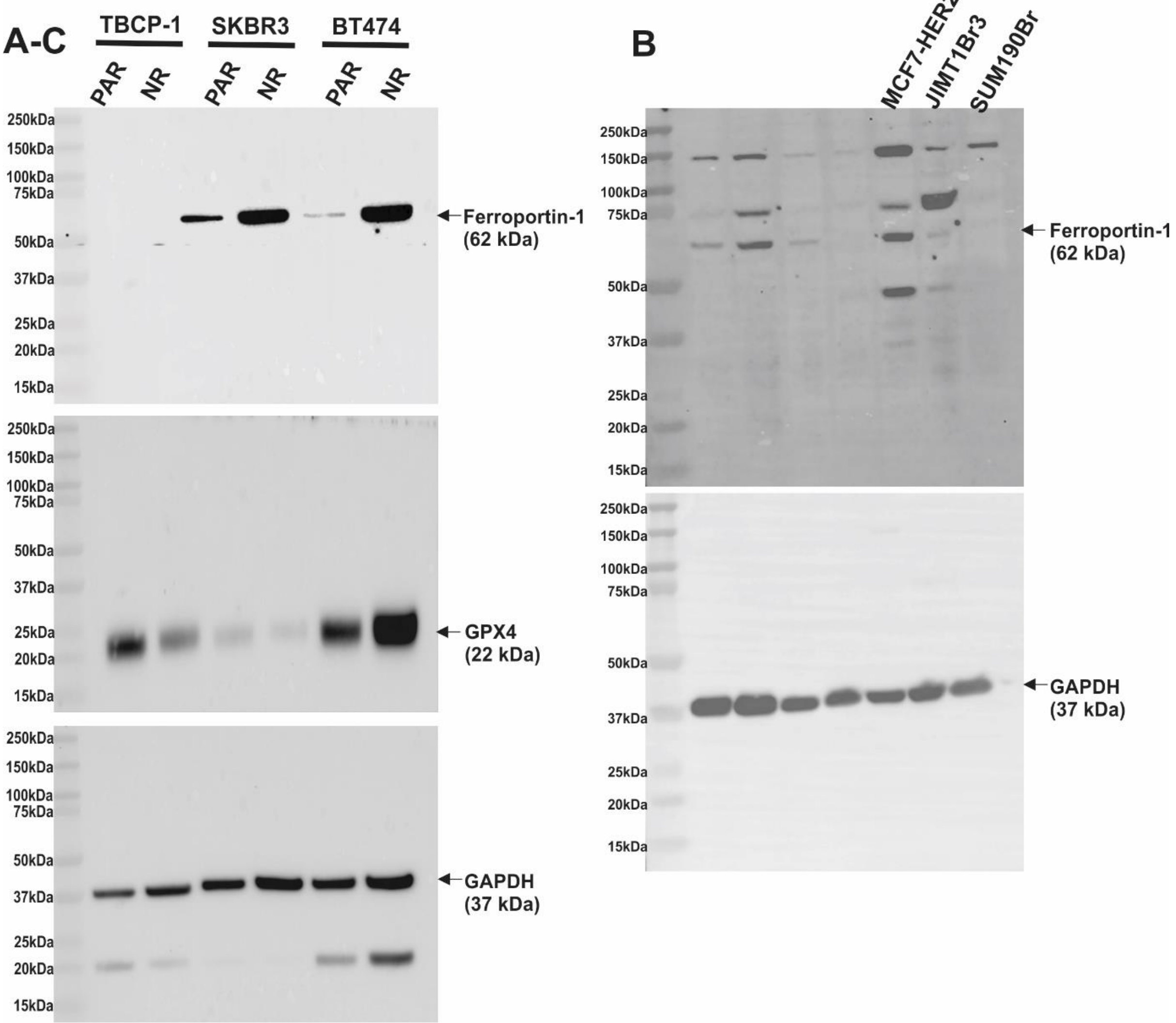

Supplementary Figure 7

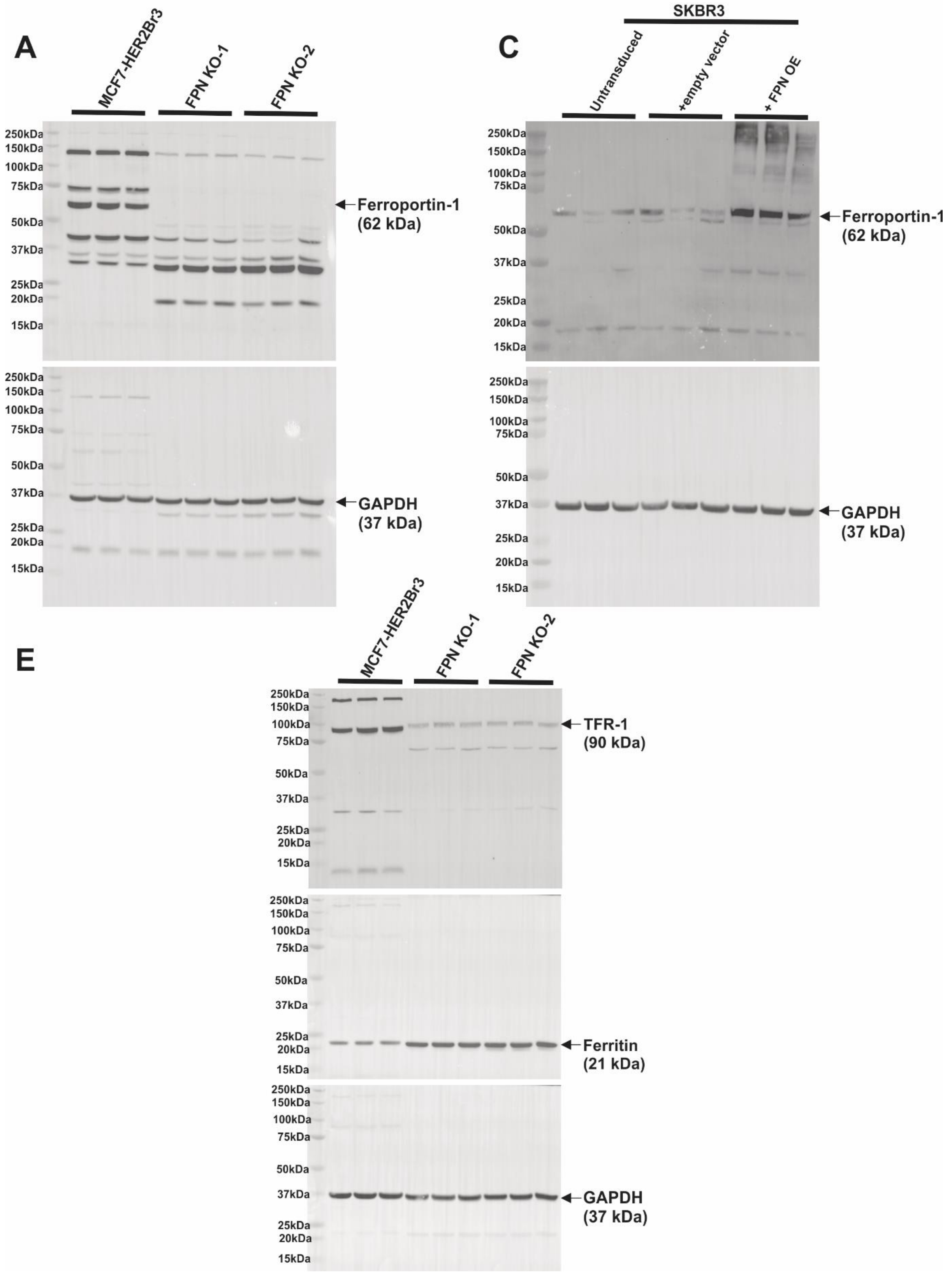

Supplement: Supplementary file 1 [file cancers-15-01216-s001.zip › cancers-2178010-File S1.pdf]
